# Metabolite ratios as potential biomarkers for type 2 diabetes: a DIRECT study

**DOI:** 10.1007/s00125-017-4436-7

**Published:** 2017-10-25

**Authors:** Sophie Molnos, Simone Wahl, Mark Haid, E. Marelise W. Eekhoff, René Pool, Anna Floegel, Joris Deelen, Daniela Much, Cornelia Prehn, Michaela Breier, Harmen H. Draisma, Nienke van Leeuwen, Annemarie M. C. Simonis-Bik, Anna Jonsson, Gonneke Willemsen, Wolfgang Bernigau, Rui Wang-Sattler, Karsten Suhre, Annette Peters, Barbara Thorand, Christian Herder, Wolfgang Rathmann, Michael Roden, Christian Gieger, Mark H. H. Kramer, Diana van Heemst, Helle K. Pedersen, Valborg Gudmundsdottir, Matthias B. Schulze, Tobias Pischon, Eco J. C. de Geus, Heiner Boeing, Dorret I. Boomsma, Anette G. Ziegler, P. Eline Slagboom, Sandra Hummel, Marian Beekman, Harald Grallert, Søren Brunak, Mark I. McCarthy, Ramneek Gupta, Ewan R. Pearson, Jerzy Adamski, Leen M. ’t Hart

**Affiliations:** 1 Research Unit of Molecular Epidemiology, Helmholtz Zentrum München, German Research Center for Environmental Health, Neuherberg, Germany; 2 Institute of Epidemiology II, Helmholtz Zentrum München, German Research Center for Environmental Health, Neuherberg, Germany; 3 German Center for Diabetes Research (DZD), München-Neuherberg, Germany; 4 Institute of Experimental Genetics, Genome Analysis Center, Helmholtz Zentrum München, German Research Center for Environmental Health, Neuherberg, Germany; 5 Department of Internal Medicine–Diabetes Center, VU University Medical Center, Amsterdam, the Netherlands; 6 Department of Biological Psychology, Vrije Universiteit, Amsterdam, the Netherlands; 7 Department of Epidemiology, German Institute of Human Nutrition Potsdam-Rehbruecke, Nuthetal, Germany; 8 Department of Molecular Epidemiology, Leiden University Medical Center, Leiden, the Netherlands; 9 Max Planck Institute for Biology of Ageing, Cologne, Germany; 10 Institute of Diabetes Research, Helmholtz Zentrum München, German Research Center for Environmental Health, Neuherberg, Germany; 11 Forschergruppe Diabetes, Klinikum rechts der Isar, Technische Universität München, Neuherberg, Germany; 12 Department of Molecular Cell Biology, Leiden University Medical Center, Albinusdreef 2, 2333ZA Leiden, the Netherlands; 13 Novo Nordisk Foundation Center for Basic Metabolic Research, Section of Metabolic Genetics, Faculty of Health and Medical Sciences, University of Copenhagen, Copenhagen, Denmark; 14 Department of Biophysics and Physiology, Weill Cornell Medical College in Qatar, Doha, Qatar; 15 Institute of Bioinformatics and Systems Biology, Helmholtz Zentrum München, German Research Center for Environmental Health, Neuherberg, Germany; 16 Institute for Clinical Diabetology, German Diabetes Center, Leibniz Center for Diabetes Research at Heinrich Heine University Düsseldorf, Düsseldorf, Germany; 17 Institute for Biometrics and Epidemiology, German Diabetes Center, Leibniz Center for Diabetes Research at Heinrich Heine University Düsseldorf, Düsseldorf, Germany; 18 Department of Endocrinology and Diabetology, Medical Faculty, Heinrich Heine University Düsseldorf, Düsseldorf, Germany; 19 Department of Gerontology and Geriatrics, Leiden University Medical Center, Leiden, the Netherlands; 20 Department of Bio and Health Informatics, Technical University of Denmark, Kongens Lyngby, Denmark; 21 Department of Molecular Epidemiology, German Institute of Human Nutrition Potsdam-Rehbruecke, Nuthetal, Germany; 22 Molecular Epidemiology Research Group, Max Delbrück Center for Molecular Medicine, Berlin Buch, Germany; 23 Oxford Centre for Diabetes, Endocrinology and Metabolism, University of Oxford, Churchill Hospital, Headington, Oxford, UK; 24 Wellcome Trust Centre for Human Genetics, University of Oxford, Oxford, UK; 25 Oxford NIHR Biomedical Research Centre, Churchill Hospital, Headington, Oxford, UK; 26 Division of Molecular and Clinical Medicine, School of Medicine, University of Dundee, Dundee, UK; 27 Institute of Experimental Genetics, Technical University of Munich, Freising-Weihenstephan, Germany; 28 Department of Epidemiology and Biostatistics, VU University Medical Center, Amsterdam, the Netherlands

**Keywords:** Epidemiology, Insulin secretion, Metabolomics, Prediction of diabetes, Type 2 diabetes

## Abstract

**Aims/hypothesis:**

Circulating metabolites have been shown to reflect metabolic changes during the development of type 2 diabetes. In this study we examined the association of metabolite levels and pairwise metabolite ratios with insulin responses after glucose, glucagon-like peptide-1 (GLP-1) and arginine stimulation. We then investigated if the identified metabolite ratios were associated with measures of OGTT-derived beta cell function and with prevalent and incident type 2 diabetes.

**Methods:**

We measured the levels of 188 metabolites in plasma samples from 130 healthy members of twin families (from the Netherlands Twin Register) at five time points during a modified 3 h hyperglycaemic clamp with glucose, GLP-1 and arginine stimulation. We validated our results in cohorts with OGTT data (*n* = 340) and epidemiological case–control studies of prevalent (*n* = 4925) and incident (*n* = 4277) diabetes. The data were analysed using regression models with adjustment for potential confounders.

**Results:**

There were dynamic changes in metabolite levels in response to the different secretagogues. Furthermore, several fasting pairwise metabolite ratios were associated with one or multiple clamp-derived measures of insulin secretion (all *p* < 9.2 × 10^−7^). These associations were significantly stronger compared with the individual metabolite components. One of the ratios, valine to phosphatidylcholine acyl-alkyl C32:2 (PC ae C32:2), in addition showed a directionally consistent positive association with OGTT-derived measures of insulin secretion and resistance (*p* ≤ 5.4 × 10^−3^) and prevalent type 2 diabetes (OR_Val_PC ae C32:2_ 2.64 [β 0.97 ± 0.09], *p* = 1.0 × 10^−27^). Furthermore, Val_PC ae C32:2 predicted incident diabetes independent of established risk factors in two epidemiological cohort studies (HR_Val_PC ae C32:2_ 1.57 [β 0.45 ± 0.06]; *p* = 1.3 × 10^−15^), leading to modest improvements in the receiver operating characteristics when added to a model containing a set of established risk factors in both cohorts (increases from 0.780 to 0.801 and from 0.862 to 0.865 respectively, when added to the model containing traditional risk factors + glucose).

**Conclusions/interpretation:**

In this study we have shown that the Val_PC ae C32:2 metabolite ratio is associated with an increased risk of type 2 diabetes and measures of insulin secretion and resistance. The observed effects were stronger than that of the individual metabolites and independent of known risk factors.

**Electronic supplementary material:**

The online version of this article (10.1007/s00125-017-4436-7) contains peer-reviewed but unedited supplementary material, which is available to authorised users.

## Introduction

Recent technological advances allow simultaneous detection of a wide range of metabolites in blood samples from healthy and diabetic individuals [1]. Studies on type 2 diabetes have provided strong evidence for the association of several blood metabolites with both prevalent and incident type 2 diabetes. In particular, the branched-chain amino acids (BCAAs; valine, leucine and isoleucine) and several phospholipids have consistently been shown to associate with disease progression [1–4]. Furthermore, there is evidence from OGTTs that these metabolites also associate with insulin secretion and/or insulin sensitivity [5–7]. However, OGTT-derived measures do not allow detailed analysis of insulin secretion, for example the response to various non-glucose insulin secretagogues such as glucagon-like peptide-1 (GLP-1) and arginine. GLP-1 is a gut hormone that stimulates insulin secretion from the pancreas, and arginine can be used as a measure of (near maximal) functional beta cell mass [8]. Alterations in the ratios between two single metabolites may point at perturbations in pathways relevant for a certain disease or phenotype and metabolite ratios are indeed known to associate with specific phenotypes [9–12]. The analysis of metabolite profiles and ratios in response to different insulin secretagogues are thus relevant for further elucidating the underlying biology of the development of type 2 diabetes. Furthermore, they may be useful for early identification of individuals with an increased risk of type 2 diabetes beyond what can be achieved with currently known risk factors.

To the best of our knowledge, this is the first study to analyse metabolite ratios in relation to insulin secretion phenotypes and type 2 diabetes risk.

## Methods

### Study design

**Fig. 1:**
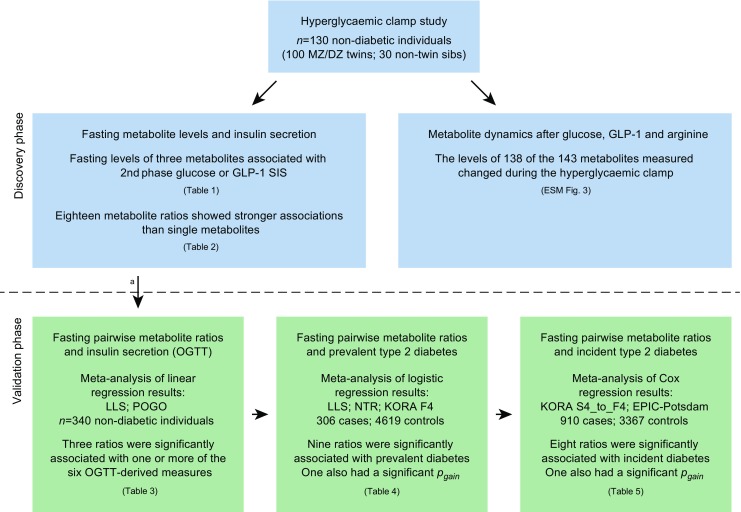
Schematic overview of the design used in the discovery (blue) and validation (green) phases of the study. MZ, monozygotic; DZ, dizygotic; sibs, siblings. Further details on the study samples can be found in ESM Methods. ^a^Most replication cohorts had only ten of the 18 ratios available

A schematic outline of the study and the rationale for selecting the cohorts is provided in Fig. 1 and in the electronic supplementary material (ESM) Methods. All studies were approved by the appropriate local institutional review boards and participants provided written informed consent before participating in the study.

### Discovery hyperglycaemic clamp study sample

**Fig. 2:**
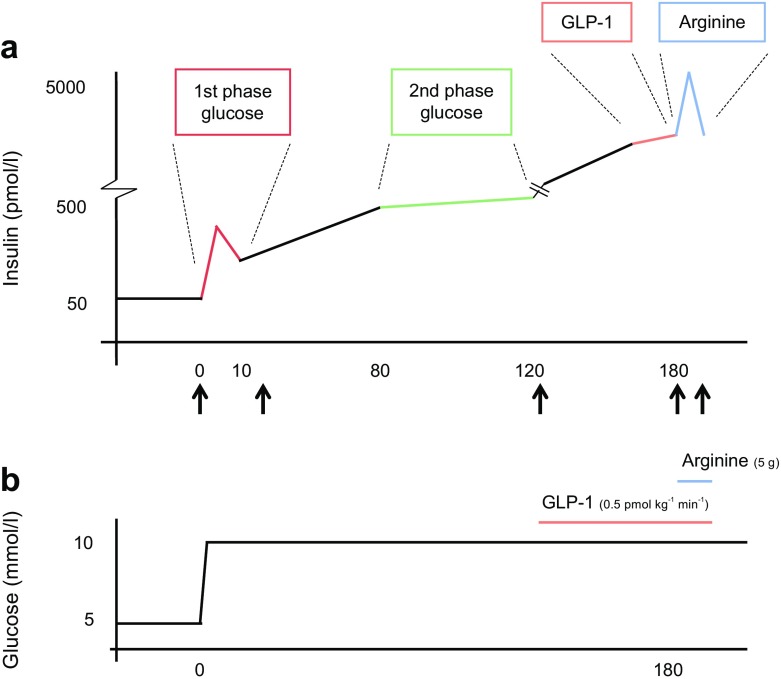
(**a**) Insulin responses. First- and second-phase GSIS (red and green, respectively), GLP-1-SIS (orange) and arginine-SIS (blue). Blood samples for metabolomics measurements were drawn at *t* = 0, 30, 120, 180 and 190 min as indicated by the black arrows. (**b**) Glucose levels. Hyperglycaemia was established and maintained at 10 mmol/l glucose via variable infusion of glucose. After 2 h, insulin secretion was further stimulated using i.v. GLP-1 infusion (1.5 pmol/kg bolus for 1 min at *t* = 120 followed by a continuous infusion of 0.5 pmol kg^−1^ min^−1^ for 1 h). The near maximal insulin response was assessed by injecting a bolus of 5 g arginine hydrochloride at *t* = 180 min

Metabolite profiles and their responses to glucose, GLP-1 and arginine stimulation were studied using a modified 3 h hyperglycaemic clamp in 130 participants of the Netherlands Twin Register (NTR) [13]. Of the 130 participants, 100 were twins and 30 were non-twin siblings from 54 families. Six of the participants had impaired glucose tolerance, while the remaining individuals had normal glucose tolerance as determined by OGTT. The clinical characteristics of the study group and details of the procedure are described in ESM Methods, ESM Table 1 and schematically presented in Figs 1, 2.

### Validation OGTT study samples

Next we validated our results in two independent cohorts with OGTT data: the Leiden Longevity Study (LLS) [14] and the POGO (Postpartum Outcomes in mothers with Gestational diabetes and their Offspring) study [15] (see ESM Methods for further details). Clinical characteristics of the study participants can be found in ESM Tables 2 and 3. From these studies we included a total of 340 non-diabetic participants who all underwent a standardised OGTT. We calculated six surrogate measures of insulin secretion and insulin resistance (ESM Table 4).

### Validation type 2 diabetes study sample

The metabolites that demonstrated significant associations in the clamp phase of the study were further investigated in four independent epidemiological studies where we studied associations with prevalent (LLS [14, 16], NTR [17, 18]; the cooperative health research in the region of Augsburg, Germany [KORA F4] study [19, 20]) or incident (KORA S4_to_F4 prospective follow-up [19, 20] and the European Prospective Investigation into Cancer and Nutrition-Potsdam [EPIC-Potsdam] study [21]) type 2 diabetes. Both the KORA S4_to_F4 and the EPIC-Potsdam studies have an average of 7 years follow-up. Further details of the studies, sampling methods and data collection can be found in references [17–21] and ESM Methods, ESM Tables 2, 5–8 and ESM Figs 1, 2. In the analysis for prevalent diabetes we included a total of 306 individuals with prevalent type 2 diabetes and 4619 non-diabetic volunteers. For the analysis of incident diabetes, we included 910 participants who were free of diabetes at baseline when blood was drawn but who developed type 2 diabetes during follow-up, and 3367 non-diabetic volunteers.

### Metabolomic measurements

Plasma concentrations of metabolites in the hyperglycaemic clamp cohort were determined with a commercial assay (Absolute*IDQ* p180 Kit; Biocrates Life Sciences, Innsbruck, Austria). The assay allows the quantification of 188 metabolites. The metabolite abbreviations are provided in ESM Table 9, metabolite naming was as described in Römisch-Margl et al [22]. Fasting and samples at four subsequent time points during the clamp (Fig. 2) were analysed according to the manufacturer’s protocol. A detailed description of the method can be found in the ESM Methods [23]. After quality control, 143 metabolites (135 metabolites and eight calculated compositions) remained for analysis. In the LLS, NTR, KORA F4 and EPIC-Potsdam cohorts, the Absolute*IDQ* p150 Kit was used, according to the methods and quality control procedures as described previously [17, 22]. ESM Table 9 describes all metabolites measured with either the p180 or p150 kits including metabolites that failed quality control in the discovery sample.

### Statistics

#### Discovery phase

In order to account for the family relationships in the hyperglycaemic clamp study we fitted generalised estimating equations (GEEs) using the R package GEEpack, v1.2-0.1 [24] (https://cran.r-project.org/web/packages/geepack/index.html). To analyse dynamic changes in metabolite levels between the different time points the linear regression models were adjusted for age, sex and BMI. In order to reduce the chance of false positives we applied stringent Bonferroni correction to correct for multiple testing (*p* ≤ 3.5 × 10^−4^; using *α* = 0.05 and 143 metabolites/tests). All six clamp-derived phenotypes were quantile normalised before analysis. To study the associations of fasting metabolites or their ratios we applied linear regression models (GEE) unadjusted, age and sex adjusted or adjusted for age, sex, BMI, glucose tolerance status, insulin sensitivity index (if relevant) as potential confounders. The Bonferroni corrected threshold was *p* ≤ 5.8 × 10^−5^ (i.e. 858 tests, 143 metabolites × six phenotypes). All possible pairwise metabolite ratios were calculated (log[metab1/metab2]) [12] and analysed as described above for single metabolites. The Bonferroni corrected threshold for the metabolite ratios was *p* ≤ 9.2 × 10^−7^ (54,270 tests, 9045 ratios × six phenotypes). In addition, the *p*_*gain*_ for each of the metabolite ratios and *p*_*gain*_ threshold was calculated (see ESM Methods for details) [12]. A *p*_*gain*_ above the threshold value suggests that the association of the metabolite ratio is stronger than that of the two individual metabolites alone.

#### Validation phase

To allow comparisons across cohorts and to facilitate meta-analysis, metabolite level data were log-transformed followed by z-scaling before analysis. Associations between OGTT-derived measures, prevalent diabetes and metabolite ratios were investigated using either linear or logistic regression models with adjustment for age, sex, BMI, use of lipid lowering medication, study-specific covariates and fasting status (where appropriate) as covariates. Only complete cases with no missing data were analysed. A fixed-effects meta-analysis was performed using the R package Meta v4.3-2 [25] (https://cran.r-project.org/web/packages/meta/index.html).

For the associations between the metabolite ratios and incident diabetes, we performed a Cox proportional hazards regression analysis with covariates as described by Wang-Sattler et al [26] and Floegel et al [7]. See ESM Table 10 for details on the covariates included. The above described base models, to which the ratio of valine and phosphatidylcholine acyl-alkyl (PC ae) C32:2 was added, reflect established prediction models which have been validated in several independent cohort studies [27–29]. We used several procedures to evaluate the accuracy of the models as described in the ESM Methods.

## Results

### Discovery phase

#### Metabolite dynamics after glucose, GLP-1 and arginine stimulation

There were many significant dynamic metabolite responses observed during the hyperglycaemic clamp procedure. Within group responses were, in general, very similar (i.e. the acylcarnitines, amino acids, etc.; ESM Fig. 3). After glucose stimulation (*t* = 30 or 120 min vs *t* = 0), we noted significant reductions (*p* ≤ 3.5 × 10^−4^) in the levels of most of the acylcarnitines (10/12), amino acids (21/21), phosphatidylcholines (68/69; except PC ae C42:0), biogenic amines (8/8) and sphingolipids (13/13). However, only a few of the lysophosphatidylcholines (4/11) changed significantly. About one-third of the metabolites that had reduced levels upon stimulation with glucose showed a further reduction after stimulation with GLP-1 (*t* = 180 vs *t* = 120). These metabolites belong to the acylcarnitines (10/12), amino acids (21/21), biogenic amines (5/8) and phosphatidylcholines (9/69). Of the metabolites that were unaffected by glucose stimulation only the acylcarnitine C0 decreased significantly after GLP-1 stimulation. After additional stimulation with arginine (*t* = 190 vs t = 180) about half of the metabolites showed a further significant change. These include acylcarnitines (4/12), amino acids (16/21), phosphatidylcholines (37/69), lysophosphatidylcholines (8/11), biogenic amines (2/8) and sphingolipids (11/13). Only four metabolites, the lysophosphatidylcholines containing myristic acid (C14:0), palmitic acid (16:0), palmitoleic acid (C16:1) and arachidonic acid (C20:4), responded exclusively to arginine stimulation, suggesting that they are specific to arginine. Remarkably, we also observed a large significant increase of phosphatidylcholine acyl-acyl (PC aa) C42:1 after arginine stimulation.

#### Fasting metabolite levels and insulin secretion (hyperglycaemic clamp)

In the remainder of the discovery study we focused on associations of baseline fasting metabolite levels and pairwise metabolite ratios with the insulin responses after stimulation with the various stimuli. Three baseline metabolites, PC aa C32:1, PC aa C34:4 and PC aa C38:5, showed a significant negative association with second-phase glucose-stimulated insulin secretion (GSIS) or GLP-1-stimulated insulin secretion (SIS) after correction for multiple testing (*p* < 5.8 × 10^−5^; Table 1). PC aa C34:4 was associated with both second-phase GSIS and GLP-1-SIS (Table 1). These associations were independent of the effects of age, sex, BMI, glucose tolerance status and insulin sensitivity. PC aa C34:4 and several other metabolites showed suggestive evidence for an association with the other phenotypes as well (*p* < 1.0 × 10^−3^; ESM Table 11).

**Table 1:** Metabolites significantly (*p* < 5.8 × 10^−5^) associated with insulin secretion measured using hyperglycaemic clamps

Phenotype	Metabolite	β (SE)	*p*
First-phase GSIS	None		
Second-phase GSIS	PC aa C34:4	−0.308 (0.073)	2.46 × 10^−5^
	PC aa C38.5	−0.023 (0.006)	3.23 × 10^−5^
	PC aa C32:1	−0.027 (0.007)	3.34 × 10^−5^
GLP-1-SIS	PC aa C34:4	−0.254 (0.060)	2.12 × 10^−5^
Arginine-SIS	None		
Disposition index	None		
Insulin sensitivity index	None		

β (SE) and *p* value were obtained from linear regressions (GEE)

Model: hyperglycaemic clamp phenotype ~ standardised metabolite level + age + sex + BMI + glucose tolerance status + insulin sensitivity (if relevant)

Eighteen fasting pairwise metabolite ratios showed associations that were significantly stronger than the individual metabolites (Table 2), i.e. having a *p*_*gain*_ above the threshold. The ratio between alanine and glycine showed the strongest association (with the insulin sensitivity index; β − 0.970 (0.145), *p* = 2.0 × 10^−11^, *p*_*gain*_ = 2.8 × 10^8^). PC aa C34:4 was the only metabolite that was significant in the single metabolite and the pairwise metabolite ratio analyses (Tables 1, 2; the results from the crude models are shown in ESM Tables 12, 13).

**Table 2:** Significant metabolite ratios (*p* < 9.2 × 10^−7^ and *p*_*gain*_ > 1350) for insulin secretion measured using hyperglycaemic clamps

Phenotype	Metabolite ratio	β (SE)	*p*	*p*_*gain*_
First-phase GSIS	None			
Second-phase GSIS	Ile_PC aa C34:3	0.793 (0.133)	2.71 × 10^−9^	8.5 × 10^4^
	Ile_PC aa C34:4	0.532 (0.093)	8.75 × 10^−9^	2811
	Val_PC aa C34:4	0.550 (0.096)	1.06 × 10^−8^	2321
	Leu_PC aa C34:3	0.785 (0.140)	2.33 × 10^−8^	9836
	Ile_PC aa C32:3	0.783 (0.141)	2.58 × 10^−8^	1.8 × 10^4^
	Ile_PC aa C36:4	0.817 (0.148)	3.34 × 10^−8^	1772
	Val_PC aa C34:3	0.804 (0.150)	8.95 × 10^−8^	2561
	Ser_PC ae C32:2	0.929 (0.179)	2.02 × 10^−7^	4918
	Val_PC ae C32:2	0.999 (0.194)	2.50 × 10^−7^	3974
	Val_PC ae C36:0	1.074 (0.210)	3.07 × 10^−7^	1.1 × 10^4^
	Gln_PC ae C32:2	0.913 (0.181)	4.20 × 10^−7^	2365
	Ile_PC ae C36:0	0.955 (0.189)	4.62 × 10^−7^	7541
GLP-1-SIS	PC aa C34:4_PC aa C38:1	−0.458 (0.080)	1.02 × 10^−8^	2078
Arginine-SIS	None			
Disposition index	PC ae C36:5_PC ae C38:4	1.569 (0.308)	3.44 × 10^−7^	3.0 × 10^4^
Insulin sensitivity index	Ala_Gly	−0.970 (0.145)	2.04 × 10^−11^	2.8 × 10^8^
	PC aa C32:3_PC ae C34:3	−1.334 (0.219)	1.07 × 10^−9^	5.4 × 10^6^
	Ala_lysoPC a C18:1	−1.102 (0.208)	1.13 × 10^−7^	1.8 × 10^4^
	Val_lysoPC a C18:1	−1.248 (0.247)	4.13 × 10^−7^	5060

β (SE) and *p* value were obtained from linear regressions (GEE)

Model: hyperglycaemic clamp phenotype ~ standardised metabolite ratio + age + sex + BMI + glucose tolerance status + insulin sensitivity (if relevant)

*p*_*gain*_ was calculated by dividing the lowest *p* value of the single metabolites by the *p* value of the ratio as described by Petersen et al [12]

lysoPC a, lysophosphatidylcholine acyl

### Validation phase

Since it was not possible to replicate our findings in cohorts with similar hyperglycaemic clamp data, we use existing metabolomics data from OGTTs to validate our findings. OGTTs are used to study insulin sensitivity and beta cell responses after stimulation with glucose. Since our main associations were with second-phase GSIS we assumed that similar associations could be found between fasting metabolite levels and insulin secretion measures as derived from OGTTs. We attempted to further validate the observed associations in various epidemiological cohort studies with type 2 diabetes as the endpoint. Most of these existing cohorts used the Biocrates Absolute*IDQ* p150 Kit measuring fewer metabolites. Therefore, a maximum of ten out of the 18 ratios could be used in the meta-analyses (ESM Table 9).

#### Fasting pairwise metabolite ratios and insulin secretion (OGTT)

In two studies, the LLS and POGO, a total of 340 participants underwent an OGTT. We focused our analyses on six commonly used OGTT-derived measures of insulin secretion and insulin resistance that were available. Analysis of the previously identified fasting metabolite ratios that could also be calculated in these cohorts showed several significant associations (ESM Tables 14, 15). After meta-analysis of the data from both OGTT studies the most significant associations were observed with the ratios of valine to PC ae C32:2, PC aa C32:3 to PC ae C34:3 and valine to lysophosphatidylcholine acyl C18:1 and target variables AUC_glucose_, AUC_insulin,_ AUC_glucose_/AUC_insulin_ and/or HOMA-IR (all *p* < 5.4 × 10^−3^; Table 3), but no associations were found with the insulinogenic index or corrected insulin response. These findings were independent of potential confounders (results from the crude models are shown in ESM Table 16). Additional adjustment for insulin sensitivity, as calculated by HOMA-IR, led to slightly weaker associations with some of the variables (ESM Table 17). However, further adjustment for fasting glucose levels did not essentially affect our results.

**Table 3:** Significant association results from a meta-analysis of OGTT data from LLS and POGO

Metabolite ratio	AUC_glucose (mmol/l × min)_		AUC_Insulin (pmol/l × min)_		AUC_Insulin_/AUC_glucose (pmol/mmol)_		Insulinogenic index		Corrected insulin response		HOMA-IR	
	β (SE)	*p* value	β (SE)	*p* value	β (SE)	*p* value	β (SE)	*p* value	β (SE)	*p* value	β (SE)	*p* value
Val_PC ae C32:2	0.103 (0.037)	5.35 × 10^−3^	0.455 (0.102)	7.76 × 10^−6^	0.345 (0.099)	5.28 × 10^−4^	−0.010 (0.134)	0.94	−0.039 (0.134)	0.77	0.466 (0.137)	6.49 × 10^−4^
PC aa C32:3_PC ae C34:3	0.025 (0.045)	0.58	0.526 (0.109)	1.33 × 10^−6^	0.458 (0.107)	1.85 × 10^−5^	0.215 (0.154)	0.16	0.235 (0.154)	0.13	0.516 (0.145)	3.75 × 10^−4^
Val_lysoPC a C18:1	0.145 (0.032)	5.00 × 10^−6^	0.538 (0.095)	1.40 × 10^−8^	0.389 (0.095)	4.30 × 10^−5^	0.142 (0.134)	0.29	0.077 (0.132)	0.56	0.528 (0.122)	1.54 × 10^−5^

Data represent β (SE) and *p* value from the meta-analysis of the individual linear regression analyses

Association of metabolite ratios significant in the discovery hyperglycaemic clamp study with OGTT-derived measures

Model: OGTT phenotype ~ standardised metabolite ratio + age + sex + BMI + lipid lowering medication + study-specific covariates

Threshold for significance, six tests *p* < 8.3 × 10^−3^

#### Fasting pairwise metabolite ratios and prevalent type 2 diabetes

Next we tested if the pairwise metabolite ratios were associated with prevalent diabetes in three independent epidemiological studies (306 diabetic and 4619 control participants). In a fixed-effects meta-analysis of fully adjusted models, we showed that nine out of the ten tested ratios were significantly associated with prevalent type 2 diabetes (Table 4, all *p* ≤ 6.4 × 10^−5^; the results for crude models are shown in ESM Table 18). Only the ratio of valine to PC ae C32:2, showing the strongest association with prevalent type 2 diabetes (OR_Val_PC ae C32:2_ 2.64 [β 0.97 ± 0.09], *p* = 1.0 × 10^−27^), showed a *p*_*gain*_ above the threshold, i.e. the effect was much stronger than that of the two individual metabolites (Table 4, ESM Table 19; both *p* ≥ 2.2 × 10^−16^, *p*_*gain*_ = 2.2 × 10^11^).

**Table 4:** Logistic regression of metabolite ratios with prevalent type 2 diabetes

Metabolite ratio	LLS		NTR		KORA F4		Meta-analysis		
	β (SE)	*p*	β (SE)	*p*	β (SE)	*p*	β (SE)	*p*	*p*_*gain*_
Ile_PC aa C34:3	na								
Ile_PC aa C34:4	na								
Val_PC aa C34:4	0.387 (0.198)	5.11 × 10^−2^	0.399 (0.160)	1.29 × 10^−2^	0.381 (0.094)	4.62 × 10^−5^	0.386 (0.075)	2.69 × 10^−7^	0
xLeu_PC aa C34:3	0.499 (0.220)	2.28 × 10^−2^	0.632 (0.180)	4.56 × 10^−4^	0.677 (0.100)	1.03 × 10^−11^	0.644 (0.081)	2.44 × 10^−15^	0
Ile_PC aa C32:3	na								
Ile_PC aa C36:4	na								
Val_PC aa C34:3	0.654 (0.238)	6.04 × 10^−3^	0.565 (0.177)	1.44 × 10^−3^	0.657 (0.107)	7.77 × 10^−10^	0.635 (0.085)	1.07 × 10^−13^	0
Ser_PC ae C32:2	0.537 (0.237)	2.34 × 10^−2^	0.227 (0.171)	0.18	0.505 (0.088)	1.11 × 10^−8^	0.456 (0.074)	8.65 × 10^−10^	0
Val_PC ae C32:2	1.022 (0.283)	2.99 × 10^−4^	0.609 (0.180)	7.10 × 10^−4^	1.100 (0.110)	2.33 × 10^−23^	0.972 (0.089)	1.01 × 10^−27^	2.2 × 10^11^
Val_PC ae C36:0	0.922 (0.255)	2.96 × 10^−4^	0.270 (0.166)	0.10	0.593 (0.101)	4.95 × 10^−9^	0.548 (0.082)	1.93 × 10^−11^	0
Gln_PC ae C32:2	0.747 (0.265)	4.82 × 10^−3^	0.221 (0.144)	0.12	0.467 (0.093)	5.46 × 10^−7^	0.423 (0.075)	1.68 × 10^−8^	0
Ile_PC ae C36:0	na								
PC aa C34:4_PC aa C38:1	−0.001 (0.223)	0.99	na		na				
Ala_Gly	na								
PC aa C32:3_PC ae C34:3	0.345 (0.199)	8.33 × 10^−2^	0.018 (0.201)	0.93	0.313 (0.081)	1.04 × 10^−4^	0.281 (0.070)	6.42 × 10^−5^	0
Ala_lysoPC a C18:1	na								
Val_lysoPC a C18:1	0.528 (0.243)	3.00 × 10^−2^	0.311 (0.174)	7.40 × 10^−2^	0.526 (0.092)	9.17 × 10^−9^	0.484 (0.077)	3.50 × 10^−10^	0
PC ae C36:5_PC ae C38:4	−0.212 (0.205)	0.30	−0.307 (0.157)	5.11 × 10^−2^	−0.193 (0.080)	1.70 × 10^−2^	−0.216 (0.067)	1.33 × 10^−3^	0

Model: Type 2 diabetes ~ standardised metabolite ratio + age + sex + BMI + lipid lowering medication + study-specific covariates

*p*_*gain*_ was calculated by dividing the lowest *p* value of the single metabolites by the *p* value of the ratio [12]

A fixed-effect meta-analysis was applied to calculate the common effect size and *p* value across the three studies

na, not available

#### Fasting pairwise metabolite ratios at baseline and incident type 2 diabetes

Meta-analysis of the Cox regression results in two independent prospective studies (910 individuals with incident type 2 diabetes and 3367 control participants), with adjustment as shown in ESM Table 10, shows a highly significant association between the ratio of valine to PC ae C32:2 and type 2 diabetes susceptibility (Table 5; HR_Val_PC ae C32:2_ 1.57 [β 0.45 ± 0.06], *p* = 1.3 × 10^−15^; the results for the crude models are shown in ESM Table 20). Again, this association was significantly stronger than that observed for the individual metabolites (Table 5, ESM Table 21; both *p* ≥ 9.2 × 10^−9^, *p*_*gain*_ = 1.3 × 10^6^). Adding glucose levels at baseline to the model only marginally affected the results and the association remained highly significant (HR_Val_PC ae C32:2_ 1.45 [β 0.37 ± 0.06], *p* = 1.4 × 10^−9^).

**Table 5:** Cox regression of metabolite ratios with incident type 2 diabetes

Metabolite ratio	KORA-S4_to_F4		EPIC-Potsdam		Meta-analysis		
	β (SE)	*p*	β (SE)	*p*	β (SE)	*p*	*p*_*gain*_
Ile_PC aa C34:3	0.309 (0.121)	1.07 × 10^−2^	na				3^a^
Ile_PC aa C34:4	0.175 (0.118)	0.14	na				0^a^
Val_PC aa C34:4	0.085 (0.114)	0.46	0.147 (0.058)	1.05 × 10^−2^	0.135 (0.051)	8.85 × 10^−3^	0
Leu_PC aa C34:3	0.211 (0.116)	7.01 × 10^−2^	na				3^a^
Ile_PC aa C32:3	0.406 (0.130)	1.80 × 10^−3^	na				19^a^
Ile_PC aa C36:4	0.210 (0.114)	6.61 × 10^−2^	na				1^a^
Val_PC aa C34:3	0.202 (0.113)	7.36 × 10^−2^	0.152 (0.054)	4.99 × 10^−3^	0.161 (0.049)	9.32 × 10^−4^	0
Ser_PC ae C32:2	−0.042 (0.108)	0.70	0.182 (0.055)	8.48 × 10^−4^	0.137 (0.049)	5.01 × 10^−3^	0
Val_PC ae C32:2	0.403 (0.132)	2.26 × 10^−3^	0.463 (0.065)	9.41 × 10^−13^	0.451 (0.058)	7.10 × 10^−15^	1.3 × 10^6^
Val_PC ae C36:0	0.184 (0.117)	0.11	0.204 (0.057)	3.77 × 10^−4^	0.151 (0.052)	3.40 × 10^−3^	0
Gln_PC ae C32:2	0.050 (0.109)	0.65	0.090 (0.044)	3.95 × 10^−2^	0.084 (0.041)	3.77 × 10^−2^	0
Ile_PC ae C36:0	0.285 (0.122)	1.92 × 10^−2^	na				2^a^
PC aa C34:4_PC aa C38:1	0.080 (0.100)	0.43	na				1^a^
Ala_Gly	0.541 (0.111)	1.11 × 10^−6^	na				378^a^
PC aa C32:3_PC ae C34:3	0.146 (0.105)	0.17	0.293 (0.054)	7.59 × 10^−8^	0.262 (0.048)	5.73 × 10^−8^	0
Ala_lysoPC a C18:1	0.395 (0.1183)	7.97 × 10^−4^	na				11^a^
Val_lysoPC a C18:1	0.271 (0.119)	2.27 × 10^−2^	0.317 (0.055)	8.24 × 10^−9^	0.309 (0.050)	5.52 × 10^−10^	65
PC ae C36:5_PC ae C38:4	0.157 (0.102)	0.13	−0.076 (0.055)	0.17	−0.023 (0.048)	0.63	0

^a^Only calculated for the KORA data

Model: Type 2 diabetes ~ standardised metabolite ratio + study-specific covariates as shown in ESM Table 10

*p*_*gain*_ was calculated by dividing the lowest *p* value of the single metabolites by the *p* value of the ratio [12]

A fixed-effect meta-analysis was applied to calculate the common effect size and *p* value

na, not available

When the valine to PC ae C32:2 ratio was added to the existing baseline prediction model comprising all established traditional risk factors (TRF+glucose) as shown in ESM Table 10, the AUC estimated from the time-dependent receiver operating characteristics improved from 0.780 to 0.801 in the KORA S4_to_F4 study (*p* = 3.2 × 10^−2^ for the ratio, ESM Table 22), which was larger than the effect of adding the two single metabolites to the model (AUC 0.793). This is also in line with the results of the net reclassification index.

In the EPIC-Potsdam study we obtained similar results for models with TRF+glucose and TRF+glucose+Val_PC ae C32:2 (0.862 and 0.865, respectively, *p* = 1.20 × 10^−8^ for the metabolite ratio). The results were largely similar for the cross-validated performance, suggesting little overfitting in the present situation with a large sample size and few added covariates (ESM Table 22).

## Discussion

In the discovery phase, we used the hyperglycaemic clamp, the gold standard for the measurement of insulin secretion [30], to study the association between baseline fasting metabolite levels, pairwise metabolite ratios and insulin response after consecutive stimulation with three different insulin secretagogues [8]. In the validation phase, we tested whether metabolite ratios identified in our clamp study were associated with insulin responses measured using OGTT data from two independent cohorts. Finally, we investigated the associations of the metabolite ratios with prevalent and incident type 2 diabetes in four independent cohorts from the Netherlands and Germany. We observed numerous dynamic metabolite responses during the clamp study reflecting the switch from beta oxidation of fatty acids and gluconeogenesis from amino acids during the overnight fast to a state of glucose oxidation during the hyperglycaemic clamp. We have shown that the ratio of valine to PC ae C32:2 is significantly positively associated with second-phase GSIS, OGTT-derived measures including HOMA-IR, and both prevalent and incident type 2 diabetes.

One limitation of this study is the relatively small sample size in the hyperglycaemic clamp part of the discovery phase, which impacts on power and reproducibility. However, we applied stringent statistical significance criteria in order to correct for multiple testing and have therefore compromised statistical power but enhanced reproducibility. Furthermore, our discovery results are corroborated in the validation phase for which we used at least two independent cohorts per phenotype studied. As described in ESM Methods the Biocrates kit used to detect the metabolites does not allow a detailed analysis of the exact lipid composition of metabolites such as PC ae C32:2. This is a limitation to the interpretation of our results (see ESM Methods for further details). Another limitation is the use of different covariates for adjusting the Cox proportional hazards regression models in the KORA S4_to_F4 and EPIC-Potsdam studies (ESM Table 10). However, both were established sets of risk factors used previously in similar metabolomic studies [7, 26] that have also been validated in external cohorts [27–29]. Furthermore, it was the aim of this study to test if metabolite ratios have an added value to these established risk factors and not to find the optimal set of predictors. Since not all covariates are available in both studies the possibilities for harmonisation of the models were limited. Despite these differences both studies yield highly comparable results, which shows the reliability of the findings. In addition, we used a cross-validation approach, which enabled us to assess the accuracy of the predictive model.

It has been shown that metabolite ratios can reveal perturbations in pathways relevant for a certain phenotype and may thus reveal stronger and more meaningful associations [31, 32], even if the mechanism is not clear. Therefore, pairwise ratios may serve as good biomarkers with predictive ability beyond that of the single constituents because noise can be reduced, increasing statistical power [12]. Valine is a BCAA, which are among the most commonly observed metabolites to be increased in type 2 diabetes and are not only responsive to glucose stimulation but also to the glucose-lowering drugs glipizide and metformin [3, 33]. Furthermore, BCAAs are associated with insulin sensitivity [34, 35] and the development of diabetes [4]. A recent Mendelian randomisation study suggested that a causal relationship exists between increased BCAA levels and type 2 diabetes risk [36]; however, it remains to be shown that PC ae C32:2 or the ratio of valine to PC ae C32:2 are also causally related to the disease, but at present there are no genetic instruments available for the latter (see ‘GWAS look-up’ in ESM Methods).

Phosphatidylcholine species, including PC ae C32:2, have been found to be associated with type 2 diabetes. However, since the phosphatidylcholines are not detected on all metabolomics platforms, replication is less frequent compared with the BCAAs [4, 6, 7, 26]. PC ae C32:2 has been shown to be associated with prevalent [6] and incident type 2 diabetes [7] and to respond to glucose stimulation during OGTT and IVGTT [37]. It is clear from our observations that the opposing effects of valine and PC ae C32:2 on insulin secretion are not simply additive, as reflected by the much stronger association of the metabolite ratio compared with the individual metabolites. According to the Human Metabolome database, PC ae C32:2 is composed of either the fatty acids C16:1/C16:1, C18:1/C14:1 or C18:2/C14:0 (www.HMDB.ca, accessed 1 October 2016) [38]. Recently, it has been shown that BCAA catabolism and lipogenesis are linked in adipose tissue [39–41]. These studies have shown that catabolism of the BCAAs (leucine, isoleucine and valine) contributes to the synthesis of odd-chain and even-chain fatty acids, such as C14, C16 and C18 chains (i.e. the constituents of PC ae C32:2). It was also shown that BCAA-derived metabolites up or downstream of the branched-chain-alpha-ketoacid dehydrogenase (BCKD) complex, being a rate-limiting step in BCAA catabolism, were associated oppositely with the risk of type 2 diabetes [36]. Further research is necessary to investigate possible functional relationships between valine and PC ae C32:2, and whether or not there is a direct causal relationship with the observed associations with GSIS and the risk of developing diabetes.

In addition to the ratio of valine to PC ae C32:2, we also note several other significant associations in our hyperglycaemic clamp experiments. For example PC aa C32:1 was associated with reduced second-phase GSIS. In previous studies by Floegel et al and Wang-Sattler et al this metabolite has been associated with an increased risk of impaired glucose tolerance and incident type 2 diabetes [7, 26]. Thus, reduced second-phase GSIS provides a potential mechanism for these previous observations. Furthermore, two other phosphatidylcholines, PC aa C34:4 and PC aa C38:5, were previously identified to be reduced in individuals with type 2 diabetes [42] or pregnant women with gestational diabetes mellitus [43]. Interestingly, these metabolites were also found to be influenced by the obesity associated variant in the *FTO* gene during OGTTs [37]. As such, our data substantiate these previous findings. We also note a significant increase in PC aa C42:1 after arginine stimulation (ESM Fig. 3). This metabolite was previously found to be decreased in individuals with type 2 diabetes [6]. Since the samples from different individuals and time points were randomised and the effect was not caused by a few individuals or outliers this seems to be a genuine observation requiring further investigation.

Next to the single metabolite associations and the valine to PC ae C32:2 ratio, the ratio of alanine and glycine strongly associated with insulin sensitivity measured using the hyperglycaemic clamp and incident diabetes in the KORA S4_to_F4 cohort. It is of interest that both amino acids have previously been identified in metabolomics studies in diabetes, indeed displaying opposing effects (reviewed in [4]). Unfortunately, alanine is not measured with the Absolute*IDQ* p150 Kit and thus the ratio could not be calculated in the other studies and as such findings could not be further validated. If validated in other studies this ratio could be of use in prediction of insulin resistance and diabetes risk.

Here we have shown that the addition of the valine to PC ae C32:2 metabolite ratio improved the accuracy of prediction of incident type 2 diabetes in a model containing known risk factors in both the KORA S4_to_F4 and EPIC-Potsdam cohorts, corroborating results from previous studies that only investigated associations with individual metabolites [7, 26]. We have also shown associations with augmented second-phase GSIS and AUC_insulin_ independent of measures of insulin resistance and other covariates (ESM Table 23). In addition, we found a positive correlation with HOMA-IR. Therefore, we speculate that the increased diabetes risk is attributable to increases in insulin resistance rather than insulin secretion, as has been suggested previously for valine and other BCAAs [34, 35]. Furthermore, our insulin secretion studies are mainly from healthy individuals and it may be that associations with augmented insulin secretion are dependent on the level of glycaemia as we have previously shown for a genetic variant of *G6PC2* [44].

It is important to note that in all of our analyses the effect of the ratio is larger than that observed with the individual metabolites suggesting that the use of ratios may improve prediction above that of the single metabolites. Large prospective studies aiming to identify the best set of predictors (including traditional risk factors and metabolites) are needed to fully elucidate the clinical applicability of using metabolite ratios in the identification of individuals at risk of developing type 2 diabetes. Since metabolomics measurements are simple and relatively non-invasive and alterations in metabolite profiles can be detected years before overt disease develops, the analysis of metabolite ratios may prove to be a useful instrument in personalising prevention and treatment strategies for type 2 diabetes.

In conclusion, we have shown that the ratio of valine to PC ae C32:2 in blood is positively associated with insulin secretion, HOMA-IR and prevalent type 2 diabetes. Furthermore, it predicts incident type 2 diabetes independent of known risk factors, suggesting that it could be useful as an early biomarker for identification of individuals at increased risk for type 2 diabetes.

## Electronic supplementary material

ESM (PDF 1216 kb)

## Abbreviations

BCAA: Branched-chain amino acids
BCKD: Branched-chain-alpha-ketoacid dehydrogenase
EPIC-Potsdam: European Prospective Investigation into Cancer and Nutrition-Potsdam
GEE: Generalised estimating equations
GLP-1: Glucagon-like peptide-1
GSIS: Glucose-stimulated insulin secretion
KORA: Cooperative health research in the region of Augsburg, Germany
LLS: Leiden Longevity Study
NTR: Netherlands Twin Register
PC aa: Phosphatidylcholine acyl-acyl
PC ae: Phosphatidylcholine acyl-alkyl
POGO: Postpartum Outcomes in mothers with Gestational diabetes and their Offspring
SIS: Stimulated insulin secretion
TRF: Traditional risk factors
