# Supplementary material for: Metabolite ratios as potential biomarkers for type 2 diabetes: a DIRECT study
Source: Diabetologia. 2017 Oct 25;61(1):117–29. doi: 10.1007/s00125-017-4436-7 (PMC6448944; doi:10.1007/s00125-017-4436-7)
Supplement: Supplementary file 1 — (PDF 1216 kb) [file 125_2017_4436_MOESM1_ESM.pdf]

ESM methods, tables and figures.

Molnos et al. **Metabolite ratios as potential biomarkers for type 2 diabetes: a DIRECT study**

ESM Methods

Design of the study

A schematic overview of the study is presented in figure 1.

Discovery hyperglycaemic clamp study sample

For the discovery study, aiming to identify metabolites and or ratios of metabolites associated with measures of insulin secretion we used healthy volunteers of the Netherlands twin register (NTR data [1]). Subjects were investigated using the modified hyperglycaemic glucose clamp, the gold standard for measuring insulin secretion [2-4].

Validation OGTT study samples

During the validation phase of the study we used various cohorts that were already available to the DIRECT consortium and in addition contacted several other cohorts with data of interest. Given that there are to our best knowledge no other cohorts available with hyperglycaemic clamp data and targeted metabolomics generated using the Biocrates platform we choose to validate our results in studies using OGTT. For this we contacted the principle investigators of the Leiden Longevity study (LLS) [5] and the Postpartum Outcomes in mothers with Gestational diabetes and their Offspring study (POGO) [6]. The LLS study (n=231) is a study investigating successful aging in nonagenarian siblings and their offspring and the partners of the offspring as population controls. The POGO study (n=116) aims to identify long-lasting changes in the maternal and fetal metabolism and microbiome, after GDM, which contribute to subsequent development of T2D and obesity. All participants in these studies underwent a standardized 75 gram OGTT. Clinical characteristics of the study participants can be found in ESM tables 2 and 3.

Validation type 2 diabetes study sample

Regarding the studies investigating the effect of the metabolites on risk of prevalent and incident diabetes we used three independent epidemiological studies in the case of prevalent diabetes; LLS [5, 7], NTR [8, 9] and the Cooperative Health Research in the Region of Augsburg, Germany study (KORA F4)[10, 11]). The Dutch LLS and NTR studies have been described above and for this study we included 42 subjects with prevalent T2D and 540 non-diabetic controls from LLS and from NTR we included 51 prevalent T2D cases and 1251 non-diabetic controls all with available Biocrates data. Details can be

found in ESM tables 2 and 5. KORA is a regional research platform for population-based surveys and subsequent follow-up studies in the fields of epidemiology, health economics, and health care research. We used subsets of the data from the survey KORA S4 (1999/2000) comprising 4261 subjects aged 25-74 years and the 7 years follow-up study KORA F4 (2006–2008) comprising 3080 participants. More precisely, from KORA F4 study 213 subjects with prevalent T2D and 2828 non-diabetic controls with Biocrates data were included. ESM Figure 1 shows the design of the KORA study.

For the studies of incident diabetes we used data from two studies; the KORA S4_to_F4 seven year prospective follow-up study [10, 11] and the European Prospective Investigation Into Cancer and Nutrition-Potsdam Study (EPIC-Potsdam study)[12]) one of the largest prospective studies of type 2 diabetes with metabolomics measured using the Biocrates platform. From KORA S4 participants were selected if they were 55 years or older without known diabetes that was validated with the OGTT [13]. Finally, 110 incident cases and 1170 control subjects were included into the analysis for the incident diabetes for whom Biocrates data was available. ESM Figure 1 shows the design of the KORA study.

The EPIC-Potsdam sample uses a case-cohort design including all incident cases (n=800) of the whole cohort (n=27548) and a randomly sample subcohort (n=2500) of which 2197 healthy controls with Biocrates data were used in the current study. ESM Figure 1 below shows schematically the design of the KORA F4 study on prevalent diabetes and the KORA S4_to_F4 study for incident diabetes. ESM Figure 2 shows the design of the EPIC-Potsdam cohort study.

Metabolomics measurements

The targeted metabolomics approach was based on LC-ESI-MS/MS and FIA-ESI-MS/MS measurements by Absolute*IDQ*™ p180 Kit (BIOCRATES Life Sciences AG, Innsbruck, Austria). The assay allows simultaneous quantification of 188 metabolites out of 10 µL plasma, and includes free carnitine, 39 acylcarnitines (Cx:y), 21 amino acids (19 proteinogenic + citrulline + ornithine), 21 biogenic amines, hexoses (sum of hexoses – about 90-95 % glucose), 90 glycerophospholipids (14 lysophosphatidylcholines (lysoPC) and 76 phosphatidylcholines (PC)), and 15 sphingolipids (SMx:y). The abbreviations Cx:y are used to describe the total number of carbons and double bonds of all chains, respectively (for more details see [14]). The method of Absolute*IDQ*™ p180 Kit has been proven to be in conformance with FDA-Guidline "Guidance for Industry - Bioanalytical Method Validation (May 2001"), which implies proof of reproducibility within a given error range. Measurements were performed as described in the manufacturer in manual UM-P180. Analytical specifications for LOD and evaluated quantification ranges, further LOD for semi quantitative measurements, identities of quantitative and

semi quantitative metabolites, specificity, potential interferences, linearity, precision and accuracy, reproducibility and stability were described in Biocrates manual AS-P180. The LODs were set to three times the values of the zero samples (PBS). The LLOQ and ULOQ were determined experimentally by Biocrates.

The assay procedures of the Absolute*IDQ*™ p180 Kit as well as the metabolite nomenclature have been described in detail previously [14, 15]. To ensure high quality data, metabolites with a coefficient of variance >15%, more than 50% missing values or more than 50% data points below the limit of detection were removed.

Sample handling was performed by a Hamilton Microlab STAR™ robot (Hamilton Bonaduz AG, Bonaduz, Switzerland) and a Ultravap nitrogen evaporator (Porvair Sciences, Leatherhead, U.K.), beside standard laboratory equipment. Mass spectrometric analyses were done on an API 4000 triple quadrupole system (Sciex Deutschland GmbH, Darmstadt, Germany) equipped with a 1200 Series HPLC (Agilent Technologies Deutschland GmbH, Böblingen, Germany) and a HTC PAL auto sampler (CTC Analytics, Zwingen, Switzerland) controlled by the software Analyst 1.6.1. Data evaluation for quantification of metabolite concentrations and quality assessment was performed with the Met*IDQ*™ software package, which is an integral part of the Absolute*IDQ*™ Kit. Internal standards serve as reference for the calculation of metabolite concentrations [µM].

Comments on PC ae 32:2

The PC ae 32:2 corresponds to HMDB13411 or 1-palmitoleyl-2-palmitoleoyl-sn-glycero-3-phosphocholine or according to IUPAC: (2-{[(2R)-3-[(9Z)-hexadec-9-en-1-yloxy]-2-[(9Z)-hexadec-9-enoyloxy]propyl phosphonato]oxy}ethyl)trimethylazanium (Figure below). However, there might be other molecular lipids being measured under the synonym "PC ae 32:2". The targeted metabolomics applied in this study does not allow for an unequivocal differentiation of isobaric species of lipids having the same total carbon number but differing in chains length or position of double bonds in the chains. This is well known limitation of the assay by Biocrates known to and accepted by epidemiologic community in studies of UK-Twins, Botnia, EPIC or KORA cohorts. We disclosed this aspect in our original publication (referenced as #25) by Römisch-Margl et al.

Figure. Structure of PC ae C32:2 and L-valine.

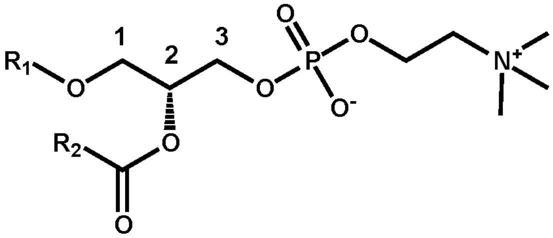

PC ae C32:2

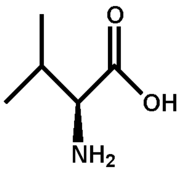

L-Valine

Chemical formulas of PC ae 32:2 and L-valine are shown. Please note that for PC ae 32:2 the carbon number is calculated as R1 + R2 +1 =32, and the double bounds number is 2 for R1 + R2. It is not known which specific chain lengths are present.

Statistics

The p_{gain} for each of the metabolite ratios and p_{gain} threshold was calculated as described previously [16]. In brief, the p_{gain} is defined as the ratio of the lowest p-value of the two individual metabolites and the p-value of the metabolite ratio. A p_{gain} above the threshold value, (B/(2*α); with B=number of metabolites tested (135) and α=0.05) suggests that the metabolite ratio carries more information than the two single metabolites.

We explored whether the associations between metabolites or ratios and incident T2D were linear or not using fractional polynomial models using R package mfp (version 1.5.2). This package selects the multiple fractional polynomial (MFP) model which best predicts the outcome. However, only linear associations were identified in KORA data.

As performance criteria, the areas under the receiver-operating characteristic curves (AUCs) were calculated using the R package *survivalROC*, v1.0.3 [17] in KORA S4_to_F4 and *PROC Logistic* in SAS statistical software (SAS, Cary, NC, USA) in EPIC-Potsdam. In KORA, two methods were applied to evaluate the accuracy of the different models: the area under the receiver operating characteristic curve (AUC) from the time-dependent ROC curve [17] and the net reclassification index (NRI) to measure the differences between two models [18]. Comparison of the new model with the standard model adjusted for traditional risk factors was done by calculating the NRI using the categories 0–3.0%, 3.1–8.0%, 8.1–15.0% and 15% [18, 19] with the R package *nricens,* version1.3 (https://cran.r-project.org/web/packages/nricens/).

The 95% CI of the time-dependent ROC curve was estimated using the empirical bootstrap method by resampling the data 5000 times and computing each time the AUC [20].

A model generally performs better in the data used to fit the model than in independent data –an issue referred to as "overfitting" [21]. Therefore, internal validation approaches are recommended to estimate *predictive performance*, or more specifically, the performance a model is expected to have in independent data drawn from the same underlying population. Here, we used 10-fold cross-validation in one of the cohorts, the KORA S4_to_F4 study [i.e., dividing data into 10 folds, and for each fold, fitting the model on the remaining 9 folds and evaluating it on the 1st fold]. The cross-validation was repeated randomly 100 times followed by averaging of the performance estimates in order to increase stability [22] using the R package *cvTools*, v0.3.2 [23].

GWAS look-up

To assess the usability of publicly available GWAS summary data on metabolites for Mendelian randomization studies into the potential causal relationship between the valine to phosphatidylcholine acyl alkyl C32:2 ratio and T2DM, we looked up the results for the single metabolites comprising this ratio as well as for the ratio itself in two sources: Supplementary Table 1 of Illig et al., Nature Genetics: doi:10.1038/ng.507 and "gwas.eu" (accessed 12 April 2017).

None of the results reported in these sources included the ratio between valine and phosphatidylcholine acyl alkyl C32:2, while the lowest P values reported in the Illig et al. Nat Genet 2010 study (their Supplementary Table 1) for the single metabolites were 2.70E-07 (SNP rs890206) and 1.44E-06 (SNP rs11144083) for valine (i.e., variable "Val PTC" in their Supplementary Table 1) and phosphatidylcholine acyl alkyl C32:2 (i.e., "PC ae C32:2" in their Supplementary Table 1), respectively. "gwas.eu" reports for valine as minimal P value 1.01E-05 (for the association with SNP rs1440581), which was found in "KORA+TwinsUK (blood)" in the Shin et al., Nat Genet. 2014, doi: 10.1038/ng.2982 study; on "gwas.eu" no results are reported for phosphatidylcholine acyl alkyl C32:2. Taken together, the two mentioned sources do not provide evidence for a genetic association with the valine_PC ae C32:2 ratio which might in turn be useful for e.g. Mendelian randomization studies as described above.

References to the ESM methods

[1] Simonis-Bik AM, Eekhoff EM, de moor MH, et al. (2009) Genetic influences on the insulin response of the beta cell to different secretagogues. Diabetologia 52: 2570-2577

[2] Fritsche A, Stefan N, Hardt E, Schutzenauer S, Haring H, Stumvoll M (2000) A novel hyperglycaemic clamp for characterization of islet function in humans: assessment of three different secretagogues, maximal insulin response and reproducibility. Eur J Clin Invest 30: 411-418

[3] Simonis-Bik AM, Nijpels G, Van Haeften TW, et al. (2010) Gene variants in the novel type 2 diabetes loci CDC123/CAMK1D, THADA, ADAMTS9, BCL11A and MTNR1B affect different aspects of pancreatic beta cell function. Diabetes 59: 293-301

[4] 't Hart LM, Simonis-Bik AM, Nijpels G, et al. (2010) A Combined Risk Allele Score of Eight Type 2 Diabetes Genes Is Associated With Reduced First Phase Glucose Stimulated Insulin Secretion During Hyperglycemic Clamps. Diabetes 59: 287-293

[5] Rozing MP, Westendorp RG, de Craen AJ, et al. (2010) Favorable glucose tolerance and lower prevalence of metabolic syndrome in offspring without diabetes mellitus of nonagenarian siblings: the Leiden longevity study. J Am Geriatr Soc 58: 564-569

[6] Hummel S, Much D, Rossbauer M, Ziegler AG, Beyerlein A (2013) Postpartum outcomes in women with gestational diabetes and their offspring: POGO study design and first-year results. The review of diabetic studies : RDS 10: 49-57

[7] Westendorp RG, van HD, Rozing MP, et al. (2009) Nonagenarian siblings and their offspring display lower risk of mortality and morbidity than sporadic nonagenarians: The Leiden Longevity Study. J Am Geriatr Soc 57: 1634-1637

[8] Draisma HH, Pool R, Kobl M, et al. (2015) Genome-wide association study identifies novel genetic variants contributing to variation in blood metabolite levels. Nature communications 6: 7208

[9] Willemsen G, de Geus EJ, Bartels M, et al. (2010) The Netherlands Twin Register biobank: a resource for genetic epidemiological studies. Twin research and human genetics : the official journal of the International Society for Twin Studies 13: 231-245

[10] Holle R, Happich M, Lowel H, Wichmann HE, Group MKS (2005) KORA--a research platform for population based health research. Gesundheitswesen 67 Suppl 1: S19-25

[11] Rathmann W, Strassburger K, Heier M, et al. (2009) Incidence of Type 2 diabetes in the elderly German population and the effect of clinical and lifestyle risk factors: KORA S4/F4 cohort study. Diabet Med 26: 1212-1219

[12] Boeing H, Korfmann A, Bergmann MM (1999) Recruitment procedures of EPIC-Germany. European Investigation into Cancer and Nutrition. Ann Nutr Metab 43: 205-215

[13] Rathmann W, Haastert B, Icks A, et al. (2003) High prevalence of undiagnosed diabetes mellitus in Southern Germany: target populations for efficient screening. The KORA survey 2000. Diabetologia 46: 182-189

[14] Römisch-Margl W, Prehn C, Bogumil R, Röhring C, Suhre K, Adamski J (2012) Procedure for tissue sample preparation and metabolite extraction for high-throughput targeted metabolomics. Metabolomics 8: 133-142

[15] Zukunft S, Sorgenfrei M, Prehn C, Möller G, Adamski J (2013) Targeted Metabolomics of Dried Blood Spot Extracts. Chromatographia 76: 1295-1305

[16] Petersen AK, Krumsiek J, Wagele B, et al. (2012) On the hypothesis-free testing of metabolite ratios in genome-wide and metabolome-wide association studies. BMC Bioinformatics 13: 120

[17] Heagerty PJ, Zheng Y (2005) Survival model predictive accuracy and ROC curves. Biometrics 61: 92-105

[18] Pencina MJ, D'Agostino RB, Sr., D'Agostino RB, Jr., Vasan RS (2008) Evaluating the added predictive ability of a new marker: from area under the ROC curve to reclassification and beyond. Statistics in medicine 27: 157-172; discussion 207-112

[19] Herder C, Baumert J, Zierer A, et al. (2011) Immunological and cardiometabolic risk factors in the prediction of type 2 diabetes and coronary events: MONICA/KORA Augsburg case-cohort study. PloS one 6: e19852
[20] Efron BT, R.J. (1994) An Introduction to the Bootstrap CRC press
[21] Hawkins DM (2004) The problem of overfitting. Journal of chemical information and computer sciences 44: 1-12
[22] Steyerberg EW, Harrell FE, Jr., Borsboom GJ, Eijkemans MJ, Vergouwe Y, Habbema JD (2001) Internal validation of predictive models: efficiency of some procedures for logistic regression analysis. J Clin Epidemiol 54: 774-781
[23] Alfons A (2012) cvTools: Cross-validation tools for regression models. package version 030

ESM Table 1. Clinical characteristics of the NTR hyperglycaemic clamp study group.

	**NTR twin cohort**
n (NGT/IGT)	124/6
Twin status (MZ, DZ, NTS)	68/32/30
Age (years)	31.4 ± 6.3
Gender (n male (%))	62 (47.7)
BMI (kg/m^2)	24.1 ± 3.5
Fasting glucose (mmol/l)	4.6 ± 0.5
2-hr glucose (mmol/l)	5.4 ± 1.2
Fasting Insulin (pmol/l)	35 (27-52)

Data are means ± SD; median (interquartile range) or number (n).
MZ monozygotic twin, DZ dizygotic twin, NTS non-twin sibling

ESM Table 2 The baseline characteristics of the LLS study population.

	**Diabetes sample**		**OGTT sample**	
Number of participants (n)	558		231	
	n missing		n missing	
Age (Years)	0	63.0 ± 6.5	0	63.2 ± 6.6
Gender (n male (%))	0	267 (47.8)	0	111 (48.1)
BMI (kg/m^2)	0	25.6 ± 3.6	0	25.4 ± 3.6
Fasting Glucose (mmol/l)	327	5.09 ± 0.50	0	5.06 ± 0.50
Fasting Insulin (pmol/l)	327	45 ± 32	0	45 ± 32
Incident Type 2 diabetes (n (%))	NA		NA	
Prevalent Type 2 diabetes (n (%))	42 (7.5)		0 (0.0)	
Lipid medication (Yes), (n (%))	47 (8.4)		11 (4.8)	
Current smoking (n (%))	76 (13.6)		21 (9.1)	
Fasting (Yes) (n (%))	239 (42.8)		231 (100)	

Data are means ± SD or number (n). NA not available.

ESM Table 3 The baseline characteristics of the POGO study population.

Number of participants (n)		116
	n missing	
Age (Years)	0	37.7 ± 5.7
Gender (n male (%))	0	0 (0.0)
BMI (kg/m^2)	0	27.8 ± 7.2
Fasting Glucose (mmol/l)	0	5.17 ± 0.77
Fasting Insulin (pmol/l)	0	57 ± 56
Incident Type 2 diabetes (n (%))		NA
Prevalent Type 2 diabetes (n (%))		7 (6.0)
Gestational Diabetes (no, %)		12 (10.3)
Lipid medication (Yes), (n (%))		NA
Current smoking (n (%))		NA
Fasting (Yes) (n (%))		116 (100)

Data are means ± SD or number (n). NA not available

ESM table 4. Calculations based on OGTT data

Measure	Formula	reference
**$AUC_{Glucose}$**	$\mathrm{AUC} = \frac{1}{2}\sum_{i=0}^{n-1}(t_{i+1} - t_i)(y_i + y_{i+1})$ t_i=time (i=0, …, i=120 mins); y_i=glucose levels at t_i	Matthews, JNS et al. BMJ 1990; 300:230-5, 1990
**$AUC_{Insulin}$**	$\mathrm{AUC} = \frac{1}{2}\sum_{i=0}^{n-1}(t_{i+1} - t_i)(y_i + y_{i+1})$ t_i=time (i=0, …, i=120 mins); y_i=insulin levels at t_i	Matthews, JNS et al. BMJ 1990; 300:230-5, 1990
**$AUC_{Insulin}$ / $AUC_{Glucose}$**	$AUC_{Insulin}$ / $AUC_{Glucose}$	
**Insulinogenic index**	($Insulin_{30\ min}$ – $Insulin_{0\ min}$) / ($Glucose_{30\ min}$ – $Glucose_{0\ min}$)	Holbrooke s. et al. JCI 1967: 323-35
**Corrected Insulin Response (CIR)**	100 × $insulin_{30\ min}$/[$glucose_{30\ min}$ × ($glucose_{30\ min}$ −3.89)]])	Sluiter WJ, et al. Diabetes 1976;25:241-4.
**HOMA-IR**	$Insulin_{0\ min\ x}$ $glucose_{0\ min}$ /22.5	Matthews DR, et al. Diabetologia 1985;28:412-19.

ESM Table 5 The baseline characteristics of the NTR type 2 diabetes study sample.

Number of participants (n)		1326
	n missing	
Age (Years)	0	51.4 ± 14.0
Gender (n male (%))	0	888 (66.7)
BMI (kg/m^2)	7	26.0 ± 3.8
Fasting Glucose (mmol/l)	1	5.71 ± 1.14
Fasting Insulin (pmol/l)	1326	NA
Incident Type 2 diabetes (n (%))		NA
Prevalent Type 2 diabetes (n (%))		51 (3.9)
Lipid medication (Yes), (n (%))		167 (12.6)
Current smoking (n (%))		NA
Fasting (Yes) (n (%))		1255 (94.7)

Data are means ± SD or number (n). NA not available

ESM Table 6 The baseline characteristics of the KORA F4 prevalent type 2 diabetes sample.

Number of participants (n)		3044
	n missing	
Age (Years)	0	56.0 ± 13.3
Gender (n male (%))	0	1472 (48.4)
BMI (kg/m^2)	15	27.6 ± 4.8
Fasting Glucose (mmol/l)	34	5.45 ± 1.05
Fasting Insulin (pmol/l)	19	54 ± 205
Incident Type 2 diabetes (n (%))		NA
Prevalent Type 2 diabetes (n (%))		213 (7.0)
Lipid medication (Yes), (n (%))		392 (12.9)
Current smoking (n (%))		466 (18.7)
Fasting (Yes) (n (%))		3026 (99.4)

Data are means ± SD or number (n). NA not available

ESM Table 7 The baseline characteristics of the KORA S4 incident type 2 diabetes sample.

Number of participants (n)		1610
	n missing	
Age (Years)	0	64.1 ± 5.5
Gender (n male (%))	0	827 (51.4)
BMI (kg/m^2)	13	28.6 ± 4.4
Fasting Glucose (mmol/l)	225	5.69 ± 0.95
Fasting Insulin (pmol/l)	144	113 ± 162
Physical activity (active)	8	670 (41.8)
Alcohol intake (g/day)	7	16±20.9
Smoking (smoker (n (%)))	2	206 (14.9)
Systolic blood pressure (mmHg)	6	136.6±20.6
HDL cholesterol (mg/dl)	1	57.5±16.4
Incident Type 2 diabetes (n (%))*		110 (9.4)
Prevalent Type 2 diabetes (n (%)) **		127 (7.9)
Lipid medication (Yes), (n (%))		195 (12.1)
Fasting (Yes) (n (%))		1349 (86.7)

Data are means ± SD or number (n). * Developed type 2 diabetes during the on average seven year follow-up from the baseline S4 measurement till the follow-up F4 measurements (denoted in the text as KORA S4_to_F4 sample including 110 incident type 2 diabetes cases and 1170 non-diabetic controls) (27).

** excluded in this study

ESM Table 8 The baseline characteristics of the EPIC-Potsdam study population.

Number of participants (n)		2997
	n missing	
Age (Years)	0	50.7 ± 8.8
Gender (n male (%))	0	1289 (43.0)
BMI (kg/m^2)	0	27.0 ± 4.7
Random Glucose (mmol/l)	0	5.14 ± 1.50
Fasting Insulin (pmol/l)	2555	53 ± 40
Coffee (cups/d)	0	2.8 ± 2.1
Whole Grain Bread (g/d)	0	44.2 ± 52.5
Red Meat (g/d)	0	44.6 ± 30.7
Waist circumference (cm)	0	88.9 ± 14.0
Incident Type 2 diabetes (n (%))		800 (26.7)
Prevalent Type 2 diabetes (n (%))		NA
Prevalent hypertension (n (%))		1630 (54.4)
Lipid medication (Yes), (n (%))		186 (6.2)
Current smoking (n (%))		617 (20.6)
Fasting (Yes) (n (%))		431 (14.3)

Data are means ± SD or number (n). NA not available.
The EPIC-Potsdam sample uses a case-cohort design including all incident cases of the whole cohort (n=27548) and a randomly sample subcohort (n=2500) of which 2197 healthy controls with Biocrates data were used in the current study (7).

ESM Table 9: List of metabolites measured with the Absolute*IDQ*tm p180 Kit
* not measured with p150 Kit; # failed quality control in the discovery cohort.

**Acylcarnitines (40)**			
C0	Carnitine	C10:1	Decenoylcarnitine
C2	Acetylcarnitine	C10:2#	Decadienylcarnitine
C3	Propionylcarnitine	C12#	Dodecanoylcarnitine
C3:1#	Propenoylcarnitine	C12:1#	Dodecenoylcarnitine
C3-OH#	Hydroxypropionylcarnitine	C12-DC#	Dodecanedioylcarnitine
C4	Butyrylcarnitine	C14#	Tetradecanoylcarnitine
C4:1#	Butenoylcarnitine	C14:1#	Tetradecenoylcarnitine
C4-OH (C3-DC)	Hydroxybutyrylcarnitine	C14:1-OH#	Hydroxytetradecenoylcarnitine
C5	Valerylcarnitine	C14:2#	Tetradecadienylcarnitine
C5:1#	Tiglylcarnitine	C14:2-OH#	Hydroxytetradecadienylcarnitine
C5:1-DC#	Glutaconylcarnitine	C16	Hexadecanoylcarnitine
C5-DC (C6-OH)#	Glutarylcarnitine (Hydroxyhexanoylcarnitine)	C16:1#	Hexadecenoylcarnitine
C5-M-DC	Methylglutarylcarnitine	C16:1-OH#	Hydroxyhexadecenoylcarnitine
C5-OH (C3-DC-M)#	Hydroxyvalerylcarnitine (Methylmalonylcarnitine)	C16:2#	Hexadecadienylcarnitine
C6 (C4:1-DC)#	Hexanoylcarnitine (Fumarylcarnitine)	C16:2-OH#	Hydroxyhexadecadienylcarnitine
C6:1#	Hexenoylcarnitine	C16-OH#	Hydroxyhexadecanoylcarnitine
C7-DC#	Pimelylcarnitine	C18#	Octadecanoylcarnitine
C8#	Octanoylcarnitine	C18:1	Octadecenoylcarnitine
C9#	Nonanoylcarnitine	C18:1-OH#	Hydroxyoctadecenoylcarnitine
C10	Decanoylcarnitine	C18:2	Octadecadienylcarnitine
**Amino Acids (21)**			
Ala*	Alanine	Lys*	Lysine
Arg	Arginine	Met	Methionine
Asn*	Asparagine	Orn	Ornithine
Asp*	Aspartate	Phe	Phenylalanine
Cit*	Citrulline	Pro	Proline
Gln	Glutamine	Ser	Serine
Glu*	Glutamate	Thr	Threonine
Gly	Glycine	Trp	Tryptophan
His	Histidine	Tyr	Tyrosine
Ile*	Isoleucine	Val	Valine
Leu*	Leucine	xLeu (p150 only)	Leucine/Isoleucine

Monosaccharides (1)
Sum of Hexoses (including Glucose)

Glycerophospholipids (90)<br>lysoPC=lysoPhosphatidylCholine; PC=PhosphatidylCholine; a=acyl; aa=diacyl; ae=acyl-alkyl)			
lysoPC a C14:0	PC aa C34:1	PC aa C42:0	PC ae C38:2
lysoPC a C16:0	PC aa C34:2	PC aa C42:1	PC ae C38:3
lysoPC a C16:1	PC aa C34:3	PC aa C42:2[#]	PC ae C38:4
lysoPC a C17:0[#]	PC aa C34:4	PC aa C42:4	PC ae C38:5
lysoPC a C18:0	PC aa C36:0	PC aa C42:5	PC ae C38:6
lysoPC a C18:1	PC aa C36:1	PC aa C42:6	PC ae C40:1
lysoPC a C18:2	PC aa C36:2	PC ae C30:0	PC ae C40:2
lysoPC a C20:3	PC aa C36:3	PC ae C30:1[#]	PC ae C40:3
lysoPC a C20:4	PC aa C36:4	PC ae C30:2[#]	PC ae C40:4
lysoPC a C24:0	PC aa C36:5	PC ae C32:1	PC ae C40:5
lysoPC a C26:0	PC aa C36:6	PC ae C32:2	PC ae C40:6
lysoPC a C26:1	PC aa C38:0	PC ae C34:0	PC ae C42:0
lysoPC a C28:0[#]	PC aa C38:1	PC ae C34:1	PC ae C42:1
lysoPC a C28:1[#]	PC aa C38:3	PC ae C34:2	PC ae C42:2
PC aa C24:0[#]	PC aa C38:4	PC ae C34:3	PC ae C42:3
PC aa C26:0	PC aa C38:5	PC ae C36:0	PC ae C42:4
PC aa C28:1	PC aa C38:6	PC ae C36:1	PC ae C42:5
PC aa C30:0	PC aa C40:1	PC ae C36:2	PC ae C44:3
PC aa C30:2[#]	PC aa C40:2	PC ae C36:3	PC ae C44:4
PC aa C32:0	PC aa C40:3	PC ae C36:4	PC ae C44:5[#]
PC aa C32:1	PC aa C40:4	PC ae C36:5	PC ae C44:6
PC aa C32:2	PC aa C40:5	PC ae C38:0	
PC aa C32:3	PC aa C40:6	PC ae C38:1[#]	

Sphingolipids (15)<br>SM=Sphingomyelin			
SM (OH) C14:1	SM C18:0	SM (OH) C22:1	SM (OH) C24:1
SM C16:0	SM C18:1	SM (OH) C22:2	SM C26:0[#]
SM C16:1	SM C20:2	SM C24:0	SM C26:1
SM (OH) C16:1	SM C22:3[#]	SM C24:1	

Biogenic Amines (21)			
Ac-Orn	Acetylornithine	PEA[,#]	Phenylethylamine
ADMA*	Asymmetric dimethylarginine	OH-Pro*[,#]	4-Hydroxyproline

alpha-AAA*	alpha-Aminoadipic acid	Putrescine*,#	Putrescine
Carnosine*,#	Carnosine	Sarcosine*	Sarcosine
Creatinine*	Creatinine	SDMA*,#	Symmetric dimethylarginine
DOPA*,#	DOPA	Serotonin*,#	Serotonin
Dopamine*,#	Dopamine	Spermidine*	Spermidine
Histamine*,#	Histamine	Spermine*,#	Spermine
Kynurenine*	Kynurenine	Taurine*	Taurine
Met-SO*,#	Methionine sulfoxide	total DMA*,#	Total dimethylarginine
Nitro-Tyr*,#	Nitrotyrosine		

ESM Table 10: Covariates used for adjustment of Cox proportional hazards regression for KORA S4/F4 and EPIC-Potsdam studies.

**Variable**	**KORA S4/F4**	**EPIC-Potsdam**
**Age**	years	years
**BMI**	kg/m^2	kg/m^2
**Sex (male/female)**	0/1	0/1
**Whole-grain bread intake**	-	g/day
**Waist circumference**	-	cm
**Physical activity**	Active/inactive	h/week
**Alcohol intake**	g/day	from beverages (non-consumers; women >0–6,6–12,and >12 g/day; and men >0–12,12–24,and >24 g/day)
**Smoking**	Smoker/non-smoker	(never, former, current ≤20 cigarettes/day, current >20 cigarettes/day)
**Education**	-	low, medium, high
**Coffee intake**	-	cups/day
**Red meat intake**	-	g/day
**Prevalent hypertension**	-	Yes/no
**Systolic blood pressure**	mm Hg	-
**Use of lipid lowering medication**	-	Yes/no
**HDL cholesterol**	Mmol/l	-
**Additional adjustment**		
**fasting glucose**	mmol/l*	Mmol/l*

Covariate selection as previously published by Wang-Sattler et al. (27) and Floegel et al. (7). Data represent the units of the included continuous covariates and or definition of categories for categorical covariates in the two studies.* only used for the model with additional adjustment for glucose.

ESM Table 11. Metabolites with $5.8x10^{-5}<p<1.0x10^{-3}$ for insulin secretion as measured with hyperglycaemic clamps.

Phenotype	Metabolite	β (SE)	*p*
1st Phase GSIS	none		
2nd Phase GSIS	PC aa C36:4	-0.006 (0.001)	$5.92x10^{-5}$
	PC aa C32:2	-0.112 (0.030)	$2.16x10^{-4}$
	PC aa C34:3	-0.047 (0.013)	$2.29x10^{-4}$
	PC aa C32:3	-2.260 (0.645)	$4.54x10^{-4}$
	PC ae C32:2	-1.775 (0.539)	$9.93x10^{-4}$
GLP-1 SIS	none		
Arginine SIS	Serine	0.010 (0.003)	$8.48x10^{-4}$
	PC aa C42:4	6.107 (1.839)	$8.99x10^{-4}$
Disposition index	none		
Insulin sensitivity index	PC aa C38:6	-0.015 (0.004)	$9.73x10^{-5}$
	lysoPC a C18:0	0.065 (0.018)	$2.79x10^{-4}$
	PC aa C40:6	-0.046 (0.014)	$7.57x10^{-4}$
	PC aa C34:4	-0.330 (0.099)	$8.33x10^{-4}$
	lysoPC a C18:2	0.034 (0.010)	$8.96x10^{-4}$

β (SE) and p-value were obtained from linear regressions (GEE). Model: hyperglycaemic clamp phenotype ~ metabolite level + age + sex + BMI + glucose tolerance status + insulin sensitivity (if relevant).

ESM Table 12. Results for unadjusted and age and sex adjusted linear regression models for the top single metabolites associated with insulin secretion as measured with hyperglycaemic clamps.

Phenotype	Metabolite	β (SE)	*p*	β (SE)	*p*
		unadjusted		***Age + sex adjusted***	
1st Phase GSIS	none				
2nd Phase GSIS	PC aa C34:4	-0.039 (0.119)	$7.43x10^{-1}$	-0.027 (0.119)	$8.22x10^{-1}$
	PC aa C38.5	-0.011 (0.011)	$2.95x10^{-1}$	-0.017 (0.011)	$1.23x10^{-1}$
	PC aa C32:1	-0.019 (0.010)	$6.36x10^{-2}$	-0.016 (0.010)	$1.10x10^{-1}$
GLP-1 SIS	PC aa C34:4	-0.271 (0.127)	$3.23x10^{-2}$	-0.111 (0.121)	$3.55x10^{-1}$
Arginine SIS	none				
Disposition index	none				
Insulin sensitivity index	none				

β (SE) and *p*-value were obtained from linear regressions (GEE). Model: hyperglycaemic clamp phenotype ~ metabolite level or hyperglycaemic clamp phenotype ~ metabolite level + age + sex.

ESM Table 13. Results for unadjusted and age and sex adjusted linear regression models for the top metabolite ratios associated with insulin secretion as measured with hyperglycaemic clamps.

Phenotype	Metabolite ratio	β (SE)	*p*	β (SE)	*p*
		unadjusted		***Age + gender adjusted***	
1st Phase GSIS	none				
2nd Phase GSIS	Ile_PC aa C34:3	0.745 (0.206)	$3.05x10^{-4}$	0.978 (0.216)	$5.90x10^{-6}$
	Ile_PC aa C34:4	0.331 (0.145)	$2.19x10^{-2}$	0.368 (0.152)	$1.54x10^{-2}$
	Val_PC aa C34:4	0.280 (0.169)	$9.75x10^{-2}$	0.290 (0.180)	$1.08x10^{-1}$
	Leu_PC aa C34:3	0.674 (0.224)	$2.67x10^{-3}$	0.908 (0.242)	$1.73x10^{-4}$
	Ile_PC aa C32:3	0.647 (0.233)	$5.60x10^{-3}$	0.747 (0.241)	$1.90x10^{-3}$
	Ile_PC aa C36:4	0.718 (0.238)	$2.58x10^{-4}$	0.914 (0.273)	$8.26x10^{-4}$
	Val_PC aa C34:3	0.719 (0.251)	$4.15x10^{-3}$	0.851 (0.260)	$1.06x10^{-3}$
	Ser_PC ae C32:2	0.932 (0.382)	$1.48x10^{-2}$	1.034 (0.386)	$7.31x10^{-3}$
	Val_PC ae C32:2	1.061 (0.297)	$3.53x10^{-4}$	1.474 (0.358)	$3.75x10^{-5}$
	Val_PC ae C36:0	1.148 (0.351)	$1.08x10^{-3}$	1.405 (0.400)	$4.37x10^{-4}$
	Gln_PC ae C32:2	0.881 (0.313)	$4.90x10^{-3}$	1.203 (0.389)	$1.99x10^{-3}$
	Ile_PC ae C36:0	1.100 (0.277)	$7.13x10^{-5}$	1.488 (0.349)	$2.03x10^{-5}$
GLP-1 SIS	PC aa C34:4_PC aa C38:1	-0.542 (0.164)	$9.76x10^{-4}$	-0.354 (0.144)	$1.41x10^{-2}$
Arginine SIS	none				
Disposition index	PC ae C36:5_PC ae C38:4	1.845 (0.395)	$3.06x10^{-6}$	1.764 (0.362)	$1.096x10^{-6}$
Insulin sensitivity index (ISI)	Ala_Gly	-1.367 (0.209)	$6.60x10^{-11}$	-1.291 (0.207)	$4.36x10^{-10}$
	PC aa C32:3_PC ae C34:3	-1.920 (0.246)	$6.15x10^{-15}$	-1.823 (0.264)	$4.90x10^{-12}$
	Ala_lysoPC a C18:1	-1.441 (0.221)	$7.62x10^{-11}$	-1.452 (0.223)	$7.52x10^{-11}$
	Val_lysoPC a C18:1	-1.670 (0.234)	$9.32x10^{-13}$	-1.665 (0.239)	$3.43x10^{-12}$

β (SE) and *p*-value were obtained from linear regressions (GEE). Model: hyperglycaemic clamp phenotype ~ metabolite level or hyperglycaemic clamp phenotype ~ metabolite level + age + sex.

ESM Table 14. Association of metabolite ratios with OGTT-derived measures in LLS study.

Metabolite ratio	$AUC_{glucose}$ (mmol/l x min)	$AUC_{Insulin}$ (pmol/l x min)	$AUC_{Insulin}$ / $AUC_{glucose}$ (mmol/lxmin / pmol/lxmin)	Insulino-genic index	CIR	HOMA-IR
	**β (SE)** ***p***	**β (SE)** ***p***	**β (SE)** ***p***	**β (SE)** ***p***	**β (SE)** ***p***	**β (SE)** ***p***
Ile_PC aa C34:3	NA					
Ile_PC aa C34:4	NA					
Val_PC aa C34:4	-0.093 (0.033) $4.49x10^{-3}$	-0.034 (0.086) 0.70	0.060 (0.084) 0.47	0.138 (0.117) 0.24	0.180 (0.119) 0.13	-0.096 (0.106) 0.37
Leu_PC aa C34:3	-0.080 (0.045) 0.047	-0.010 (0.102) 0.92	0.071 (0.095) 0.46	0.055 (0.154) 0.72	0.112 (0.146) 0.44	0.019 (0.132) 0.88
Ile_PC aa C32:3	NA					
Ile_PC aa C36:4	NA					
Val_PC aa C34:3	-0.094 (0.041) 0.022	-0.052 (0.101) 0.61	0.044 (0.097) 0.65	0.009 (0.165) 0.96	0.077 (0.152) 0.61	-0.031 (0.133) 0.81
Ser_PC ae C32:2	0.018 (0.053) 0.74	-0.058 (0.136) 0.67	-0.076 (0.125) 0.54	-0.178 (0.180) 0.32	-0.190 (0.174) 0.28	0.249 (0.146) 0.089
Val_PC ae C32:2	0.127 (0.047) $6.39x10^{-3}$	0.422 (0.119) $3.96x10^{-4}$	0.295 (0.116) 0.011	-0.046 (0.151) 0.76	-0.088 (0.150) 0.56	0.525 (0.151) $6.1x10^{-4}$
Val_PC ae C36:0	0.032 (0.044) 0.47	0.270 (0.116) 0.020	0.241 (0.118) 0.041	0.046 (0.169) 0.79	0.065 (0.162) 0.69	0.314 (0.145) 0.031
Gln_PC ae C32:2	0.072 (0.055) 0.20	0.188 (0.149) 0.21	0.116 (0.140) 0.41	-0.141 (0.180) 0.44	-0.162 (0.181) 0.37	0.319 (0.175) 0.70
Ile_PC ae C36:0	NA					
PC aa C34:4_PC aa C38:1	0.098 (0.021) $2.00x10^{-6}$	0.127 (0.067) 0.057	0.029 (0.066) 0.66	-0.135 (0.083) 0.10	-0.178 (0.082) 0.030	0.167 (0.073) 0.023
Ala_Gly	NA					
PC aa C32:3_PC ae C34:3	0.101 (0.051) 0.045	0.528 (0.130) $4.85x10^{-5}$	0.426 (0.128) $9.02x10^{-4}$	0.125 (0.181) 0.49	0.110 (0.180) 0.54	0.502 (0.163) $2.3x10^{-3}$
Ala_lysoPC a C18:1	NA					
Val_lysoPC a C18:1	0.148 (0.043) $5.95x10^{-4}$	0.668 (0.115) $5.70x10^{-9}$	0.522 (0.116) $6.98x10^{-6}$	0.315 (0.163) 0.053	0.227 (0.157) 0.15	0.646 (0.138) $4.9x10^{-6}$
PC ae C36:5_PC ae C38:4	-0.068 (0.080) 0.39	-0.127 (0.193) 0.51	-0.062 (0.178) 0.73	0.193 (0.259) 0.45	0.041 (0.253) 0.87	-0.050 (.215) 0.82

**With HOMA-IR correction***	**$AUC_{glucose}$ (mmol/l xmin)**	**$AUC_{Insulin}$ (pmol/l xmin)**	**$AUC_{Insulin}$ / $AUC_{glucose}$**	**Insulino-genic index**	**CIR**	
Val_PC aa C34:4	-0.087 (0.033) $8.58x10^{-3}$	0.006 (0.061) 0.93	0.093 (0.063) 0.14	.165 (0.108) 0.128	0.200 (0.111) 0.072	
Val_PC ae C32:2	0.090 (0.045) 0.048	0.173 (0.092) 0.059	0.084 (0.099) 0.39	-0.224 (0.156) 0.15	-0.227 (0.151) 0.13	
PC aa C34:4_PC aa C38:1	0.087 (0.021) $2.83x10^{-5}$	0.050 (0.051) 0.32	-0.037 (0.051) 0.47	-0.190 (0.075) 0.011	-0.221 (0.076) $3.42x10^{-3}$	
PC aa C32:3_PC ae C34:3	0.055 (0.051) 0.28	0.235 (0.111) 0.035	0.179 (0.118) 0.13	-0.081 (0.190) 0.67	-0.048 (0.185) 0.80	
Val_lysoPC a C18:1	0.102 (0.045) 0.022	0.363 (0.083) $1.23x10^{-5}$	0.264 (0.094) $5.08x10^{-3}$	0.102 (0.171) 0.55	0.062 (0.161) 0.70	
**With HOMA-IR + glucose correction***	**$AUC_{glucose}$ (mmol/l xmin)**	**$AUC_{Insulin}$ (pmol/l xmin)**	**$AUC_{Insulin}$ / $AUC_{glucose}$**	**Insulino-genic index**	**CIR**	
Val_PC aa C34:4	-0.063 (0.029) 0.031	0.004 (0.061) 0.945	0.068 (0.061) 0.265	0.117 (0.108) 0.282	0.121 (0.102) 0.233	
Val_PC ae C32:2	0.083 (0.046) 0.075	0.174 (0.092) 0.058	0.092 (0.095) 0.332	-0.210 (0.161) 0.193	-0.204 (0.148) 0.169	
PC aa C34:4_PC aa C38:1	0.064 (0.020) $1.38x10^{-3}$	0.053 (0.051) 0.30	-0.011 (0.050) 0.818	-0.144 (0.073) 0.049	-0.144 (0.069) 0.035	
PC aa C32:3_PC ae C34:3	0.097 (0.046) 0.033	0.234 (0.110) 0.033	0.136 (0.109) 0.214	-0.166 (0.185) 0.370	-0.185 (0.161) 0.250	
Val_lysoPC a C18:1	0.090 (0.040) 0.026	0.364 (0.083) $1.20x10^{-5}$	0.277 (0.089) $1.81x10^{-3}$	0.127 (0.165) 0.44	0.103 (0.140) 0.460	

Data were adjusted for age, sex, BMI, group status and lipid lowering medication and familial relationships (GEE). β (SE) are from LN transformed and normalized data. AUC area under the curve; CIR corrected insulin response. NA not available. * only shown for significant associations.

ESM Table 15. Association of metabolite ratios with OGTT derived measures in POGO study.

**Metabolite ratio**	**$AUC_{glucose}$** (mmol/l xmin)	**$AUC_{Insulin}$** (pmol/l xmin)	**$AUC_{Insulin}$ / $AUC_{glucose}$** (mmol/lxmin / pmol/lxmin)	**Insulinogenic index**	**CIR**	**HOMA-IR**
	**β (SE)** ***p***	**β (SE)** ***p***	**β (SE)** ***p***	**β (SE)** ***p***	**β (SE)** ***p***	**β (SE)** ***p***
Ile_PC aa C34:3	0.041 (0.045) 0.367	-0.090 (0.154) 0.560	-0.136 (0.149) 0.363	-0.293 (0.223) 0.192	-0.280 (0.225) 0.217	-0.429 (0.241) 0.078
Ile_PC aa C34:4	0.049 (0.038) 0.204	-0.060 (0.129) 0.640	-0.110 (0.125) 0.383	-0.226 (0.189) 0.235	-0.152 (0.190) 0.426	-0.271 (0.202) 0.182
Val_PC aa C34:4	0.052 (0.039) 0.182	-0.122 (0.130) 0.350	-0.178 (0.126) 0.162	-0.255 (0.190) 0.181	-0.237 (0.191) 0.218	-0.303 (0.204) 0.139
Leu_PC aa C34:3	0.025 (0.045) 0.583	-0.164 (0.151) 0.281	-0.195 (0.147) 0.188	-0.218 (0.220) 0.325	-0.272 (0.223) 0.225	-0.500 (0.237) 0.037
Ile_PC aa C32:3	0.099 (0.051) 0.057	0.168 (0.173) 0.332	0.069 (0.169) 0.685	-0.292 (0.257) 0.258	-0.246 (0.257) 0.341	-0.331 (0.277) 0.234
Ile_PC aa C36:4	0.017 (0.057) 0.76	0.110 (0.191) 0.565	0.094 (0.187) 0.615	0.087 (0.278) 0.755	-0.054 (0.283) 0.850	0.036 (0.302) 0.906
Val_PC aa C34:3	0.044 (0.046) 0.337	-0.176 (0.154) 0.257	-0.230 (0.150) 0.128	-0.327 (0.222) 0.143	-0.396 (0.225) 0.080	-0.467 (0.241) 0.055
Ser_PC ae C32:2	-0.078 (0.051) 0.131	0.205 (0.175) 0.244	0.285 (0.170) 0.096	0.391 (0.252) 0.124	-0.478 (0.255) 0.064	-0.133 (0.276) 0.632
Val_PC ae C32:2	0.064 (0.060) 0.290	0.544 (0.196) $6.63x10^{-3}$	0.482 (0.193) 0.014	0.126 (0.294) 0.669	0.156 (0.299) 0.603	0.199 (0.322) 0.538
Val_PC ae C36:0	0.105 (0.063) 0.097	0.212 (0.211) 0.318	0.100 (0.207) 0.629	-0.170 (0.309) 0.583	-0.118 (0.313) 0.71	0.060 (0.338) 0.86
Gln_PC ae C32:2	-0.047 (0.058) 0.419	0.330 (0.196) 0.095	0.396 (0.190) 0.039	0.363 (0.292) 0.217	0.549 (0.288) 0.059	0.007 (0.315) 0.981
Ile_PC ae C36:0	0.084 (0.058) 0.149	0.310 (0.192) 0.109	0.226 (0.188) 0.232	-0.084 (0.285) 0.769	0.083 (0.288) 0.774	0.109 (0.310) 0.725
PC aa C34:4_PC aa C38:1	0.000 (0.037) 0.995	0.409 (0.119) $8.78x10^{-4}$	0.420 (0.116) $7.29x10^{-3}$	0.339 (0.182) 0.065	0.331 (0.184) 0.075	0.576 (0.189) $2.90x10^{-3}$
Ala.Gly	0.068 (0.043) 0.117	0.317 (0.144) 0.030	0.255 (0.141) 0.074	0.036 (0.220) 0.870	0.135 (0.218) 0.537	0.548 (0.226) 0.017
PC aa C32:3_PC ae	-0.002 (0.061)	0.522 (0.199)	0.530 (0.194)	0.447 (0.291)	0.570 (0.295)	0.576 (0.317)

C34:3	0.974	0.010	$8.78x10^{-3}$	0.128	0.056	0.072
Ala_lysoPC a C18:1	0.044 (0.044) 0.313	0.323 (0.151) 0.035	0.287 (0.147) 0.054	0.087 (0.216) 0.688	0.228 (0.221) 0.305	0.174 (0.237) 0.466
Val_lysoPC a C18:1	0.141 (0.047) $3.40x10^{-3}$	0.262 (0.168) 0.123	0.117 (0.166) 0.483	-0.228 (0.238) 0.341	-0.284 (0.243) 0.244	0.101 (0.263) 0.702
PC ae C36:5_PC ae C38:4	0.069 (0.086) 0.426	-0.355 (0.298) 0.236	-0.428 (0.290) 0.143	0.021 (0.420) 0.961	-0.334 (0.431) 0.440	0.280 (.457) 0.541
**With HOMA-IR correction***	**$AUC_{glucose}$** (mmol/l xmin)	**$AUC_{Insulin}$** (pmol/l xmin)	**$AUC_{Insulin}$ / $AUC_{glucose}$**	**Insulinogenic index**	**CIR**	
Val_PC aa C34:4	0.060 (0.038) 0.115	-0.090 (0.122) 0.462	-0.152 (0.121) 0.213	-0.250 (0.189) 0.188	-0.226 (0.192) 0.240	
Val_PC ae C32:2	0.036 (0.006) 0.546	0.425 (0.187) 0.025	0.386 (0.188) 0.042	0.029 (0.299) 0.923	0 .105 (0.303) 0.730	
PC aa C34:4_PC aa C38:1	-0.033 (0.038) 0.395	0.298 (0.118) 0.013	0.336 (0.117) $4.88x10^{-3}$	0.282 (0.188) 0.136	0.299 (0.191) 0.120	
PC aa C32:3_PC ae C34:3	-0.043 (0.062) 0.485	0.322 (0.197) 0.105	0.375 (0.195) 0.058	0.350 (0.301) 0.247	0.521 (0.307) 0.093	
Val_lysoPC a C18:1	0.136 (0.047) $4.4x10^{-3}$	0.223 (0.157) 0.158	0.085 (0.158) 0.594	-0.253 (0.237) 0.287	-0.294 (0.243) 0.228	
**With HOMA-IR and glucose correction***	**$AUC_{glucose}$** (mmol/l xmin)	**$AUC_{Insulin}$** (pmol/l xmin)	**$AUC_{Insulin}$ / $AUC_{glucose}$**	**Insulinogenic index**	**CIR**	
Val_PC aa C34:4	0.053 (0.038) 0.158	-0.073 (0.122) 0.549	-0.126 (0.120) 0.299	-0.251 (0.190) 0.189	-0.176 (0.186) 0.347	
Val_PC ae C32:2	0.036 (0.006) 0.548	0.430 (0.187) 0.023	0.394 (0.185) 0.036	0.029 (0.301) 0.924	0 .110 (0.293) 0.707	
PC aa C34:4_PC aa C38:1	-0.025 (0.038) 0.514	0.287 (0.118) 0.017	0.316 (0.116) $7.53x10^{-3}$	0.285 (0.190) 0.137	0.244 (0.186) 0.193	
PC aa C32:3_PC ae C34:3	-0.024 (0.062) 0.704	0.289 (0.200) 0.151	0.318 (0.197) 0.109	0.356 (0.305) 0.246	0.387 (0.302) 0.204	
Val_lysoPC a C18:1	0.139 (0.0467) $3.24x10^{-3}$	0.227 (0.157) 0.151	0.091 (0.156) 0.563	-0.255 (0.238) 0.288	-0.311 (0.234) 0.188	

Data were adjusted for age, BMI, group status GDM and glucose tolerance status. β (SE) are from LN transformed and normalized data. AUC area under the curve; CIR corrected insulin response. * only shown for significant associations.

ESM Table 16. Meta-analysis results for age and sex adjusted linear regression models for the top metabolite ratios and OGTT data from LLS and POGO cohorts.

**Metabolite ratio**	**$AUC_{glucose}$ (mmol/l x min)**	**$AUC_{Insulin}$ (pmol/l x min)**	**$AUC_{Insulin}$ / $AUC_{glucose}$ (mmol/lxmin / pmol/lxmin)**	**Insulino - genic index**	**CIR**	**HOMA-IR**
	**β (SE)** ***p***	**β (SE)** ***p***	**β (SE)** ***p***	**β (SE)** ***p***	**β (SE)** ***p***	**β (SE)** ***p***
Val_PC ae C32:2	0.209 (0.039) $5.91x10^{-8}$	0.720 (0.102) $2.15x10^{-12}$	0.515 (0.096) $8.96x10^{-8}$	0.062 (0.136) 0.65	0.022 (0.134) 0.87	0.861 (0.142) $1.17x10^{-9}$
PC aa C32:3_ PC ae C34:3	0.178 (0.045) $7.56*10^{-5}$	0.744 (0.117) $2.40x10^{-10}$	0.570 (0.108) $1.39x10^{-7}$	0.154 (0.156) 0.32	0.190 (0.154) 0.22	0.854 (0.161) $1.06x10^{-7}$
Val_lysoPC a C18:1	0.222 (0.032) $2.92x10^{-12}$	0.787 (0.087) $1.38x10^{-19}$	0.561 (0.086) $6.60x10^{-11}$	0.240 (0.129) $6.28x10^{-2}$	0.178 (0.121) 0.14	0.931 (0.120) $1.13x10^{-14}$

Association of metabolite ratios significant in the discovery hyperglycaemic clamp study with OGTT derived measures. Model: OGTT phenotype ~ standardized metabolite ratio + age + sex (LLS only). Data represent β (SE), *p*-value from meta-analysis of the individual linear regression analyses.

ESM Table 17. Meta-analysis LLS + POGO OGTT data (significant ratios only).

**Metabolite ratio**	**$AUC_{glucose}$** (mmol/l x min)	**$AUC_{Insulin}$** (pmol/l x min)	**$AUC_{Insulin}$ / $AUC_{glucose}$** (mmol/lxmin / pmol/lxmin)	**Insulino - genic index**	**CIR**
**With additional HOMA-IR correction**	**β (SE)** ***p***	**β (SE)** ***p***	**β (SE)** ***p***	**β (SE)** ***p***	**β (SE)** ***p***
Val_PC ae C32:2	0.071 (0.036) 0.050	0.222 (0.083) $7.1x10^{-3}$	0.150 (0.088) 0.088	-0.170 (0.138) 0.22	-0.161 (0.135) 0.23
PC aa C32:3_PC ae C34:3	0.012 (0.049) 0.80	0.256 (0.097) $8.12x10^{-3}$	0.232 (0.101) 0.022	0.042 (0.161) 0.79	0.104 (0.158) 0.51
Val_lysoPC a C18:1	0.118 (0.033) $2.74x10^{-4}$	0.332 (0.073) $5.89x10^{-6}$	0.217 (0.081) $7.17x10^{-3}$	-0.020 (0.139) 0.89	-0.047 (0.134) 0.73
**With additional HOMA-IR + glucose correction**					
Val_PC ae C32:2	0.065 (0.036) 0.072	0.224 (0.083) $6.7x10^{-3}$	0.155 (0.085) 0.067	-0.157 (0.142) 0.27	-0.140 (0.132) 0.29
PC aa C32:3_PC ae C34:3	0.044 (0.060) 0.468	0.247 (0.096) 0.010	0.179 (0.095) 0.061	-0.026 (0.158) 0.87	-0.058 (0.142) 0.68
Val_lysoPC a C18:1	0.111 (0.030) $2.67x10^{-4}$	0.334 (0.073) $5.29x10^{-6}$	0.231 (0.077) $2.8x10^{-3}$	0.003 (0.136) 0.98	-0.006 (0.120) 0.96

Association of metabolite ratios from the hyperglycaemic clamp study with OGTT derived measures in the meta-analysis of LLS and POGO. AUC area under the curve; CIR corrected insulin response. Data represent β (SE) after meta-analysis of individual studies beta and SE from linear regression.

ESM Table 18. Results for age and sex adjusted logistic regression models for the top metabolite ratios and prevalent type 2 diabetes.

	LLS	NTR	KORA F4	Meta-analysis
**Metabolite ratio**	**β (SE)** ***p***	**β (SE)** ***p***	**β (SE)** ***p***	**β (SE)** ***p***
Ile_PC aa C34:3	na			
Ile_PC aa C34:4	na			
Val_PC aa C34:4	0.315 (0.184) $8.71x10^{-2}$	0.539 (0.149) $2.99x10^{-4}$	0.491 (0.087) $1.93x10^{-8}$	0.476 (0.070) $8.66x10^{-12}$
Leu_PC aa C34:3	na			
Ile_PC aa C32:3	na			
Ile_PC aa C36:4	na			
Val_PC aa C34:3	0.720 (0.219) $9.93x10^{-4}$	0.951 (0.176) $6.97x10^{-8}$	0.910 (0.100) $1.18x10^{-19}$	0.893 (0.081) $2.89x10^{-28}$
Ser_PC ae C32:2	0.517 (0.211) $1.43x10^{-2}$	0.466 (0.166) $4.91x10^{-3}$	0.634 (0.084) $5.65x10^{-14}$	0.590 (0.071) $7.95x10^{-17}$
Val_PC ae C32:2	1.222 (0.251) $1.08x10^{-6}$	1.018 (0.180) $1.64x10^{-8}$	1.313 (0.105) $8.08x10^{-36}$	1.236 (0.085) $1.48x10^{-47}$
Val_PC ae C36:0	0.938 (0.220) $2.04x10^{-5}$	0.621 (0.169) $2.42x10^{-4}$	0.804 (0.098) $2.97x10^{-16}$	0.781 (0.079) $6.86x10^{-23}$
Gln_PC ae C32:2	0.763 (0.221) $5.68x10^{-5}$	0.383 (0.174) $2.75x10^{-2}$	0.611 (0.088) $3.91x10^{-12}$	0.587 (0.074) $2.27x10^{-15}$
Ile_PC ae C36:0	na			
PC aa C34:4_PC aa C38:1	0.190 (0.181) 0.29	na	na	
Ala_Gly	na			
PC aa C32:3_PC ae C34:3	0.622 (0.172) $3.12x10^{-4}$	0.439 (0.173) $1.10x10^{-2}$	0.486 (0.076) $1.77x10^{-10}$	0.498 (0.065) $1.24x10^{-14}$
Ala_lysoPC a C18:1	na			
Val_lysoPC a C18:1	0.723 (0.215) $7.55x10^{-4}$	0.653 (0.142) $4.07x10^{-6}$	0.699 (0.084) $8.44x10^{-17}$	0.691 (0.068) $6.47x10^{-24}$
PC ae C36:5_PC ae C38:4	-0.226 (0.179) 0.21	-0.422 (0.145) $3.57x10^{-3}$	-0.199 (0.075) $8.22x10^{-3}$	-0.244 (0.063) $9.80x10^{-5}$

Model: Type 2 diabetes ~ standardized metabolite ratio + age + sex + study specific covariates. Fixed effect meta-analysis was applied to calculate the common effect size and *p*-value across the three studies. na not available.

ESM Table 19. logistic regression of standardized metabolite levels with prevalent type 2 diabetes.

	LLS	NTR	KORA F4	Meta-analysis
**Metabolite**	**β (SE)** ***p***	**β (SE)** ***p***	**β (SE)** ***p***	**β (SE)** ***p***
H1	0.766 (0.166) $4.03x10^{-6}$	1.915 (0.240) $4.62x10^{-16}$	1.258 (0.083) $2.08x10^{-52}$	1.226 (0.071) $6.06x10^{-67}$
Ala	n a			
Ile	n a			
xLeu*	0.120 (0.156) 0.44	0.426 (0.135) $1.56x10^{-3}$	0.744 (0.086) $6.26x10^{-18}$	0.558 (0.066) $2.29x10^{-17}$
Gln	-0.166 (0.184) 0.37	0.067 (0.147) 0.65	-0.244 (0.085) $3.87x10^{-3}$	-0.166 (0.068) 0.015
Gly	-0.286 (0.256) 0.26	-0.536 (0.245) 0.028	-0.340 (0.101) $7.16x10^{-4}$	-0.359 (0.088) $4.31x10^{-5}$
Ser	-0.137 (0.198) 0.49	0.057 (0.150) 0.70	-0.044 (0.084) 0.61	-0.034 (0.069) 0.62
Val	0.309 (0.166) 0.063	0.410 (0.133) $1.96x10^{-3}$	0.753 (0.096) $5.06x10^{-15}$	0.577 (0.070) $2,22x10^{-16}$
PC aa C32:3	-0.649 (0.299) 0.030	-0.423 (0.206) 0.040	-0.312 (0.093) $8.15x10^{-4}$	-0.354 (0.082) $1.38x10^{-5}$
PC aa C34:3	-0.441 (0.311) 0.16	-0.222 (0.211) 0.29	-0.180 (0.098) 0.065	-0.207 (0.085) 0.016
PC aa C34:4	-0.176 (0.248) 0.48	-0.153 (0.172) 0.37	-0.042 (0.087) 0.63	-0.089 (0.072) 0.22
PC aa C38:1	-0.228 (0.187) 0.22	n a	n a	
PC ae C32:2	-0.884 (0.330) $7.34x10^{-3}$	-0.329 (0.211) 0.12	-0.717 (0.103) $3.76x10^{-12}$	-0.660 (0.089) $1,31x10^{-13}$
PC ae C34:3	-0.923 (0.363) 0.011	-0.222 (0.222) 0.32	-0.550 (0.102) $7.66x10^{-8}$	-0.519 (0.090) $7,43x10^{-9}$
PC ae C36:0	-0.698 (0.315) 0.027	0.000 (0.180) 0.998	-0.150 (0.083) 0.072	-0.155 (0.073) 0.035
PC ae C36:5	-0.496 (0.295) 0.092	-0.434 (0.178) 0.015	-0.390 (0.090) $1.38x10^{-5}$	-0.406 (0.077) $1.60x10^{-7}$
PC ae C38:4	-0.359 (0.223) 0.11	-0.267 (0.147) 0.070	-0.279 (0.091) $2.11x10^{-3}$	-0.285 (0.073) $9.87x10^{-5}$
LysoPC a C18:1	-0.277 (0.297) 0.35	-0.057 (0.187) 0.76	-0.143 (0.092) 0.12	-0.137 (0.080) 0.085

Model: Type 2 diabetes ~ standardized metabolite concentration + age + sex + BMI (kg/m^2) + lipid lowering medication + study specific covariates. * The Absolute*IDQ*^{tm} p150 kit does not distinguish between Leucine and isoleucine, xLeu represents the combined levels of both leucine and isoleucine.

ESM Table 20. Results for age and sex adjusted Cox regression models for the top metabolite ratios and incident type 2 diabetes.

	KORA-S4/F4	EPIC-Potsdam	Meta-analysis
**Metabolite ratio**	**β (SE)**<br>***p***	**β (SE)**<br>***p***	**β (SE)**<br>***p***
Ile_PC aa C34:3	0.548 (0.112)<br>$9.94x10^{-7}$	na	
Ile_PC aa C34:4	0.369 (0.109)<br>$6.82x10^{-4}$	na	
Val_PC aa C34:4	0.288 (0.103)<br>$5.07x10^{-3}$	0.128 (0.047)<br>$6.10x10^{-3}$	0.156 (0.043)<br>$2.74x10^{-4}$
Leu_PC aa C34:3	0.424 (0.109)<br>$9.25x10^{-5}$	na	
Ile_PC aa C32:3	0.688 (0.117)<br>$4.48x10^{-9}$	na	
Ile_PC aa C36:4	0.390 (0.108)<br>$3.14x10^{-4}$	na	
Val_PC aa C34:3	0.404 (0.104)<br>$2.19x10^{-5}$	0.219 (0.046)<br>$2.03x10^{-6}$	0.249 (0.042)<br>$3.12x10^{-9}$
Ser_PC ae C32:2	0.149 (0.100)<br>0.13	0.146 (0.045)<br>$1.10x10^{-3}$	0.147 (0.041)<br>$3.57x10^{-4}$
Val_PC ae C32:2	0.666 (0.113)<br>$4.10x10^{-9}$	0.655 (0.054)<br>$1.06x10^{-33}$	0.657 (0.049)<br>$1.92x10^{-41}$
Val_PC ae C36:0	0.394 (0.105)<br>$1.70x10^{-4}$	0.319 (0.050)<br>$1.89x10^{-10}$	0.333 (0.045)<br>$1.66x10^{-13}$
Gln_PC ae C32:2	0.181 (0.102)<br>$7.65x10^{-2}$	0.098 (0.038)<br>$1.02x10^{-2}$	0.108 (0.036)<br>$2.40x10^{-3}$
Ile_PC ae C36:0	0.475 (0.109)<br>$1.37x10^{-5}$	na	
PC aa C34:4_PC aa C38:1	0.036 (0.096)<br>0.71	na	
Ala_Gly	0.695 (0.109)<br>$1.64x10^{-10}$	na	
PC aa C32:3_PC ae C34:3	0.321 (0.099)<br>$1.16x10^{-3}$	0.462 (0.049)<br>$7.97x10^{-21}$	0.434 (0.044)<br>$4.69x10^{-23}$
Ala_lysoPC a C18:1	0.612 (0.105)<br>$5.62x10^{-9}$	na	
Val_lysoPC a C18:1	0.535 (0.099)<br>$6.95x10^{-8}$	0.526 (0.048)<br>$2.45x10^{-28}$	0.528 (0.043)<br>$2.49x10^{-34}$
PC ae C36:5_PC ae C38:4	0.124 (0.096)<br>0.20	0.049 (0.045)<br>0.28	.063 (0.041)<br>0.12

Model: Type 2 diabetes ~ standardized metabolite ratio + age + sex. Fixed effect meta-analysis was applied to calculate the common effect size and *p*-value. na not available.

ESM Table 21. Cox regression of standardized metabolite levels with incident type 2 diabetes in EPIC-Potsdam and KORA S4_to_F4.

	**KORA S4_to_F4**	**EPIC-Potsdam**	**Meta-analysis**
**Metabolite**	**β (SE)**<br>***p***	**β (SE)**<br>***p***	**β (SE)**<br>***p***
H1	0.896 (0.084)<br>$3.00x10^{-26}$	0.674 (0.056)<br>$3.46x10^{-33}$	0.741 (0.046)<br>$2.04x10^{-57}$
Ala	0.247 (0.094)<br>$9.03x10^{-3}$	n a	
Ile	0.220 (0.104)<br>$3.39x10^{-2}$	n a	
Leu	0.133 (0.104)<br>0.20	n a	
Gln	-0.229 (0.106)<br>3.11x10-2	-0.177 (0.051)<br>$4.72x10^{-4}$	-0.187 (0.046)<br>$4.38x10^{-05}$
Gly	-0.449 (0.127)<br>$4.20x10^{-4}$	-0.301 (0.063)<br>$1.84x10^{-6}$	-0.330 (0.056)<br>$5.02x10^{-9}$
Ser	-0.298 (0.109)<br>$6.27x10^{-3}$	-0.030 (0.057)<br>0.59	-0.087 (0.050)<br>$8.25x10^{-2}$
Val	0.132 (0.105)<br>0.21	0.298 (0.054)<br>$3.29x10^{-8}$	0.263 (0.048)<br>$3.57x10^{-8}$
PC aa C32:3	-0.263 (0.124)<br>$3.42x10^{-2}$	-0.108 (0.056)<br>0.055	-0.135 (0.051)<br>$8.79x10^{-3}$
PC aa C34:3	-0.104 (0.109)<br>0.34	0.050 (0.053)<br>0.35	0.021 (0.048)<br>0.66
PC aa C34:4	0.026 (0.114)<br>0.82	0.060 (0.057)<br>0.29	0.053 (0.051)<br>0.30
PC aa C38:1	-0.098 (0.102)<br>0.33	n a	
PC ae C32:2	-0.469 (0.141)<br>$8.74x10^{-4}$	-0.275 (0.057)<br>$1.30x10^{-6}$	-0.302 (0.053)<br>$9.16x10^{-9}$
PC ae C34:3	-0.531 (0.144)<br>$2.20x10^{-4}$	-0.452 (0.064)<br>$1.12x10^{-12}$	-0.465 (0.058)<br>$1.22x10^{-15}$
PC ae C36:0	-0.059 (0.114)<br>0.60	0.038 (0.049)<br>0.44	0.023 (0.045)<br>0.62
PC ae C36:5	-0.025 (0.112)<br>0.82	-0.156 (0.051)<br>$2.39x10^{-3}$	-0.125 (0.047)<br>$7.63x10^{-3}$
PC ae C38:4	-0.139 (0.114)<br>0.22	-0.117 (0.054)<br>$3.03x10^{-2}$	-0.121 (0.049)<br>$1.32x10^{-2}$
LysoPC a C18:1	-0.205 (0.123)<br>$9.61x10^{-2}$	-0.176 (0.057)<br>$2.09x10^{-3}$	-0.181 (0.052)<br>$4.81x10^{-4}$

Model: Type 2 diabetes = standardized metabolite concentration + age + sex + BMI (kg/m^2) + lipid lowering medication + study specific covariates

ESM Table 22. Apparent and cross validated model performance for incident diabetes in KORA S4/F4 and EPIC-Potsdam.

	KORA S4_to_F4					EPIC-Potsdam		
**Model**	**AUC_ROC (95% CI)**	**NRI**	**β (SE)***	**p**		**AUC_ROC (95% CI)**	**β (SE)***	**p**
**Val_PC ae C32:2**	0.697 (0.637- 0.752)	-0.206	0.651 (0.102)	$2.05x10^{-10}$		0.674 (0.652-0.695)	0.683 (0.050)	$5.27x10^{-4}$
CV result	0.693	-0.193						
**Glucose + Val_PC ae C32:2**	0.782 (0.725-0.847)	0.036	0.562 (0.113)	$6.43x10^{-7}$		0.771 (0.751-0.790)	0.560 (0.054)	$6.15x10^{-25}$
CV result	0.779	0.047						
**Glucose + TRF**	0.780 (0.731-0.854)	Ref	NA	NA		0.862 (0.848-0.877)	NA	NA
CV result	0.766	Ref						
**Glucose + TRF + Val + PC ae C32:2**	0.793 (0.737-0.861)	-0.016	Val: 0.05 (0.12) PC ae C32:2: -0.48 (0.15)	Val: 0.66 PC ae C32:2: $2.1x10^{-3}$ Joint effect: $8.8x10^{-3}$		0.865 (0.851-0.880)	Val: 0.49 (0.10) PC ae C32:2: - 0.32 (0.08)	Val: $2.9x10^{-7}$ PC ae C32:2: $5.5x10^{-5}$
CV result	0.774	-0.004						
**Glucose + TRF + Val_PC ae C32:2**	0.801 (0.747- 0.863)	0.013	0.311 (0.145)	$3.19x10^{-2}$		0.865 (0.851-0.880)	0.384 (0.067)	$1.20x10^{-8}$
CV result	0.781	0.019						

TRF = traditional risk factors as mentioned in ESM Table 10. CV = cross validation * β's (SE) and p-values are provided for the ratio of valine and PC ae C32:2. NA not available. NRI was calculated comparing the model in the current row and the model Glucose + TRF.

ESM Table 23. Associations of valine, PC ae C32:2 and their ratio with various measures of insulin secretion as measured with the hyperglycaemic clamp.

	**β (SE)**	**p**	**β (SE)**	**p**	**β (SE)**	**p**
	**Valine**		**PC ae C32:2**		**Val_PC ae C32:2**	
1st phase GSIS	0.005 (0.002)	$6.90x10^{-3}$	-0.077 (0.672)	0.909	0.569 (0.218)	$9.09x10^{-3}$
2nd phase GSIS	0.005 (0.002)	$7.65x10^{-3}$	-1.775 (0.539)	$9.93x10^{-4}$	0.999 (0.194)	$2.50x10^{-7}$
GLP1 SIS	0.003 (0.002)	0.083	-0.697 (0.557)	0.21	0.526 (0.308)	0.088
ARG SIS	0.002 (0.002)	0.44	0.726 (0.641)	0.257	-0.185 (0.339)	0.567
Insulin sensitivity index (ISI)	-0.003 (0.002)	0.101	1.033 (0.986)	0.249	-0.526 (0.315)	0.094
Disposition index	0.004 (0.002)	0.137	0.494 (0.822)	0.547	0.167 (0.304)	0.582

β (SE) and p-value were obtained from linear regressions (GEE). Model: hyperglycaemic clamp phenotype ~ metabolite level + age + sex + BMI + glucose tolerance status and ISI (where appropriate).

ESM Figure 1. Schematic design of the KORA F4 (left) and KORA S4_to_F4 studies.

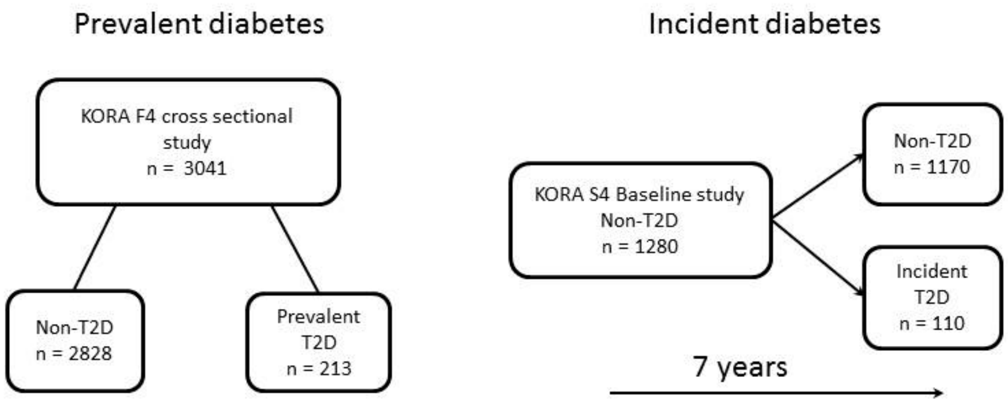

Design of the validation studies in the KORA F4 and KORA S4-to_F4 studies.

ESM Figure 2. Schematic design of the EPIC-Potsdam study.

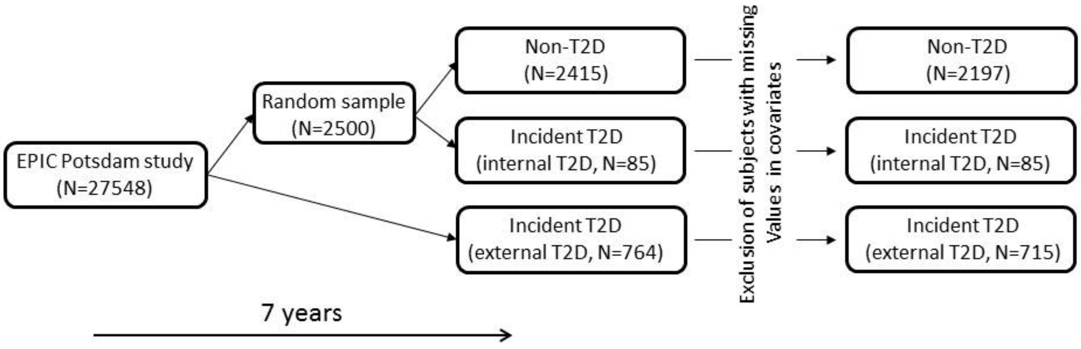

Design of the EPIC-Potsdam validation study.

ESM Figure 3. Dynamic metabolite responses during the hyperglycemic clamp procedure.

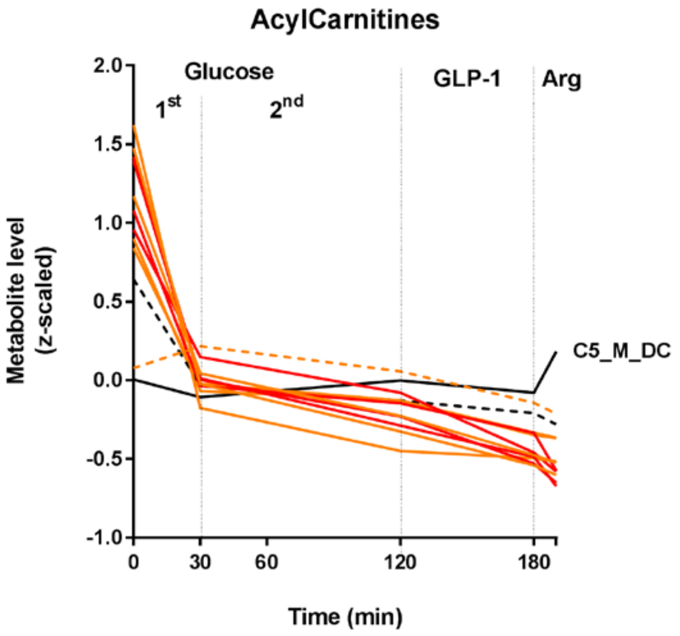

C0, C2, C3, C4, C4_OH_(C3_DC), C5, C5_M_DC, C10, C10.1, C16, C18.1, C18.2

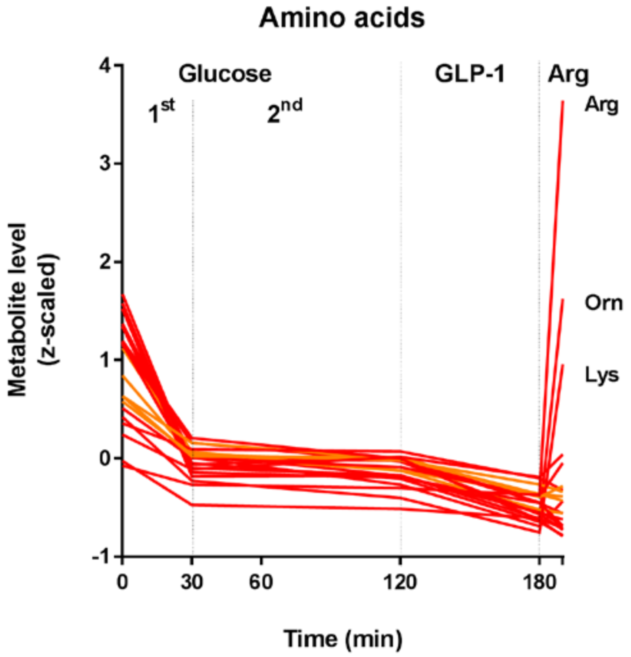

Ala, Arg, Asn, Asp, Cit, Gln, Glu, Gly, His, Ile, Leu, Lys, Met, Orn, Phe, Pro, Ser, Thr, Trp, Tyr, Val

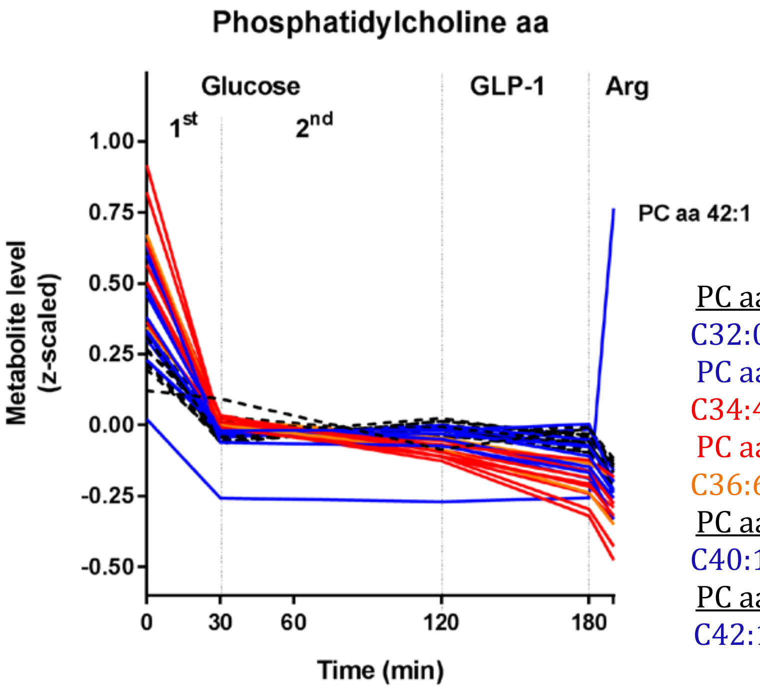

PC aa C26:0, PC aa C28:1, PC aa C30:0, PC aa C32:0, PC aa C32:1, PC aa C32:2, PC aa C32:3, PC aa C34:1, PC aa C34:2, PC aa C34:3, PC aa C34:4, PC aa C36:0, PC aa C36:1, PC aa C36:2, PC aa C36:3, PC aa C36:4, PC aa C36:5, PC aa C36:6, PC aa C38:0, PC aa C38:1, PC aa C38:3, PC aa C38:4, PC aa C38:5, PC aa C38:6, PC aa C40:1, PC aa C40:2, PC aa C40:3, PC aa C40:4, PC aa C40:5, PC aa C40:6, PC aa C42:0, PC aa C42:1, PC aa C42:4, PC aa C42:5, PC aa C42:6

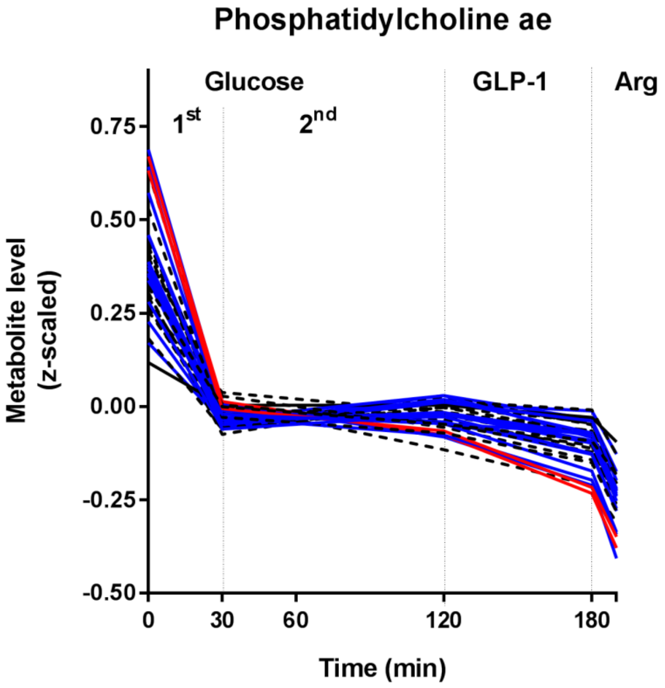

PC ae C30:0, PC ae C32:1, PC ae C32:2, PC ae C34:0, PC ae C34:1, PC ae C34:2, PC ae C34:3, PC ae C36:0, PC ae C36:1, PC ae C36:2, PC ae C36:3, PC ae C36:4, PC ae C36:5, PC ae C38:0, PC ae C38:2, PC ae C38:3, PC ae C38:4, PC ae C38:5, PC ae C38:6, PC ae C40:1, PC ae C40:2, PC ae C40:3, PC ae C40:4, PC ae C40:5, PC ae C40:6, PC ae C42:0, PC ae C42:1, PC ae C42:2, PC ae C42:3, PC ae C42:4, PC ae C42:5, PC ae C44:3, PC ae C44:4, PC ae C44:6

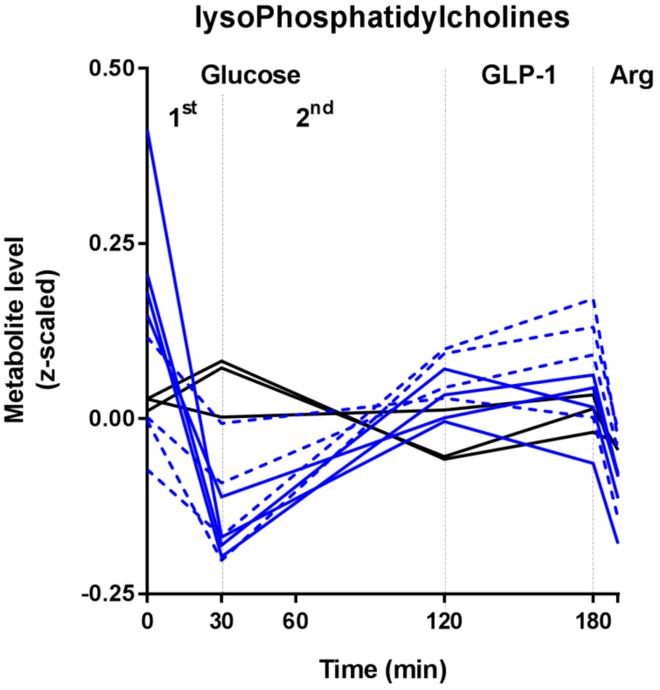

lysoPC a C14:0, lysoPC a C16:0, lysoPC a C16:1, lysoPC a C18:0, lysoPC a C18:1, lysoPC a C18:2, lysoPC a C20:3, lysoPC a C20:4, lysoPC a C24:0, lysoPC a C26:0, lysoPC a C26:1

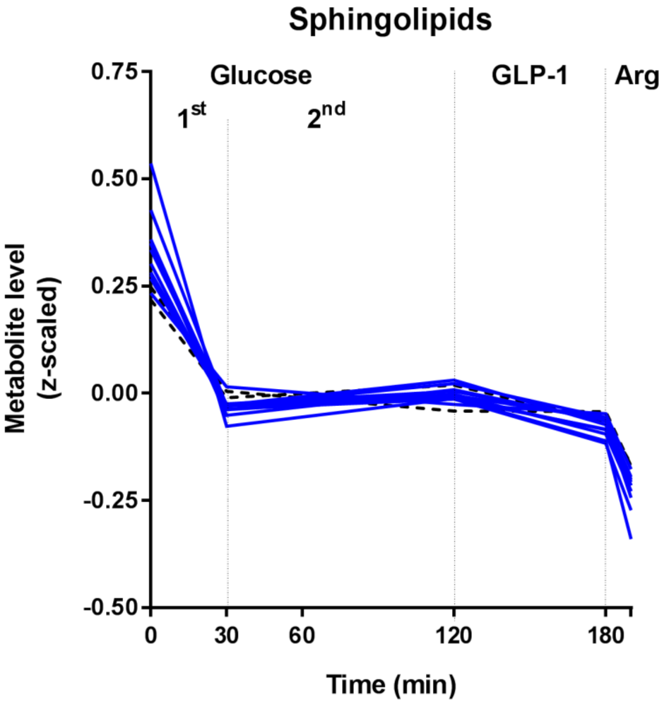

SM (OH) C14.1, SM (OH) C16.1, SM (OH) C22.1, SM (OH) C22.2, SM (OH) C24.1, SM C16.0,
SM C16.1, SM C18.0, SM C18.1, SM C20.2, SM C24.0, SM C24.1, SM C26.1

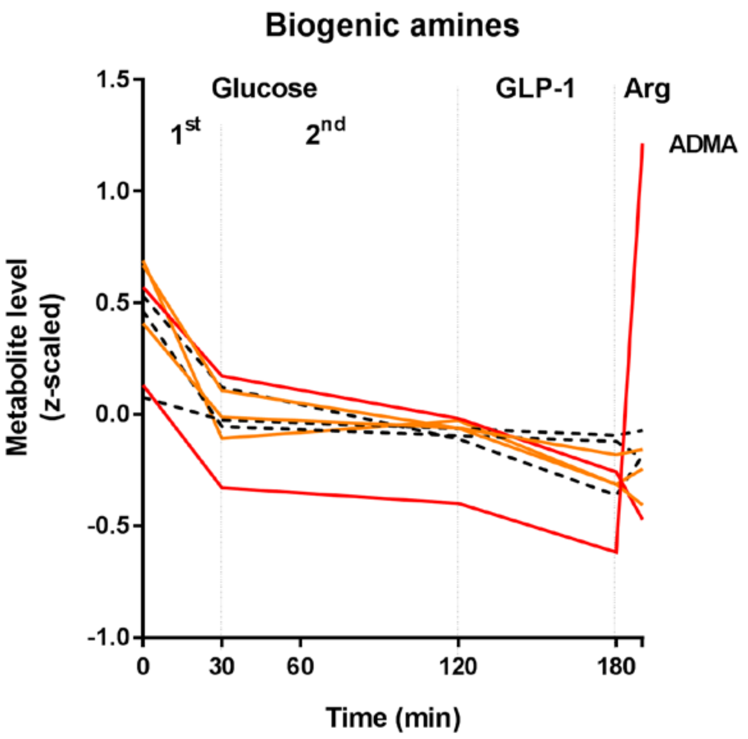

Ac-Ornitine, ADMA, alpha_AAA, Creatinine, Kynurenine, Sarcosine, Spermidine, Taurine

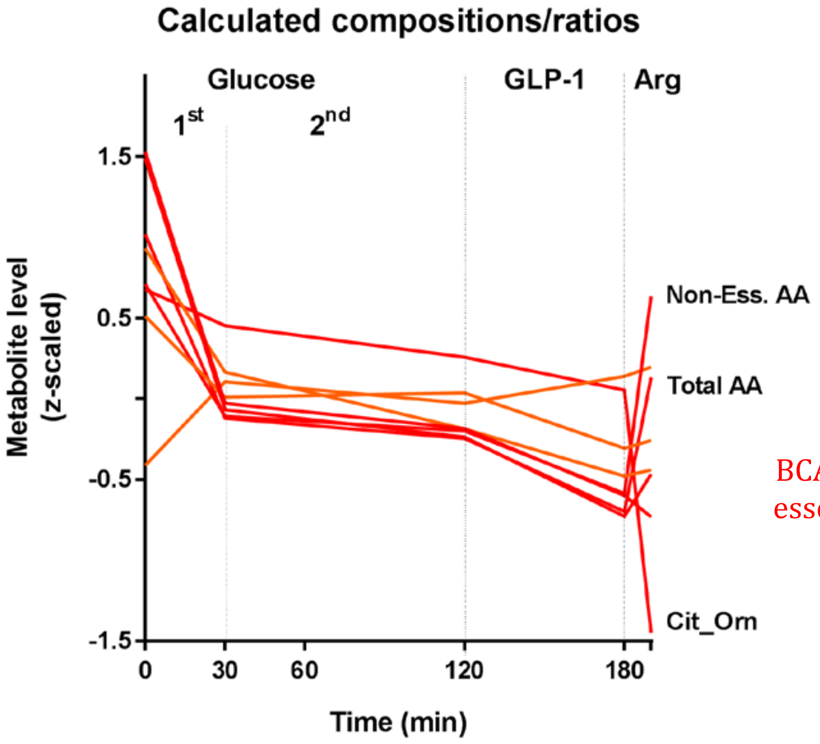

BCAA, Fisher ratio, Essential AA, non-essential AA, Glucogenic AA, Total AA, Kynurenine_Trp, Cit_Orn

Legend

black = non-significant for changes for all stimuli

red = significant changes for all stimuli

Orange = significant changes after glucose and GLP-1 stimulation

Blue = significant changes after glucose and arginine stimulation

– – – black dashes = significant changes after glucose stimulation only (underlined metabolites)

– – – Orange dashes = significant changes after GLP-1 stimulation only (underlined metabolites)

– – – Blue dashes = significant changes after arginine stimulation only (underlined metabolites)
